# 5,7,3ʹ,4ʹ-Tetrahydroxyflav-2-en-3-ol 3-*O*-glucoside, a new biosynthetic precursor of cyanidin 3-*O*-glucoside in the seed coat of black soybean, *Glycine max*

**DOI:** 10.1038/s41598-020-74098-6

**Published:** 2020-10-14

**Authors:** Kumi Yoshida, Yada Teppabut, Reo Sawaguchi, Yuhsuke Nakane, Emi Hayashi, Kin-ichi Oyama, Yuzo Nishizaki, Yukihiro Goda, Tadao Kondo

**Affiliations:** 1 Graduate School of Informatics, Nagoya University, Chikusa, Nagoya 464-8601 Japan; 2 Graduate School of Information Science, Nagoya University, Chikusa, Nagoya 464-8601 Japan; 3 Research Center for Materials Science, Nagoya University, Chikusa, Nagoya 464-8602 Japan; 4 National Institute of Health Sciences, 3-25-26 Tonomachi, Kawasaki-ku, Kawasaki, Kanagawa 210-9501 Japan

**Keywords:** Organic chemistry, Natural products, Plant sciences

## Abstract

The seed coat of mature black soybean, *Glycine max*, accumulates a high amount of cyanidin 3-*O*-glucoside (Cy3G), which is the most abundant anthocyanin in nature. In the pod, it takes two months for the seed coat color change from green to black. However, immature green beans rapidly adopt a black color within one day when the shell is removed. We analyzed the components involved in the color change of the seed coat and detected a new precursor of Cy3G, namely 5,7,3ʹ,4ʹ-tetrahydroxyflav-2-en-3-ol 3-*O*-glucoside (2F3G). Through quantitative analysis using purified and synthetic standard compounds, it was clarified that during this rapid color change, an increase in the Cy3G content was observed along with the corresponding decrease in the 2F3G content. Chemical conversion from 2F3G to Cy3G at pH 5 with air and ferrous ion was observed. Our findings allowed us to propose a new biosynthetic pathway of Cy3G via a colorless glucosylated compound, 2F3G, which was oxidized to give Cy3G.

## Introduction

Anthocyanins are plant pigments which are often responsible for the colors of flowers, fruits, vegetables, and roots, with such colors ranging widely from red through purple to blue^1–4^. In recent years, the medicinal properties of these pigments have attracted significant attention in terms of their therapeutic value for the treatment of metabolic disorder-related syndromes in humans^5–10^. Anthocyanins are known to exhibit a vast structural diversity through glycosylation and acylation, and among them, cyanidin 3-*O*-glucoside (Cy3G, **1**) is one of the simplest and most widely distributed pigments in nature, being found in cherry blossom, autumn red leaves, red-colored fruits, and the seed coat of the black soybean, *Glycine max*^3,11–14^. Furthermore, the biosynthetic pathway of Cy3G (**1**) has been well studied, and almost all genes and enzymes involved have been identified^15–20^.

**Figure 1:**
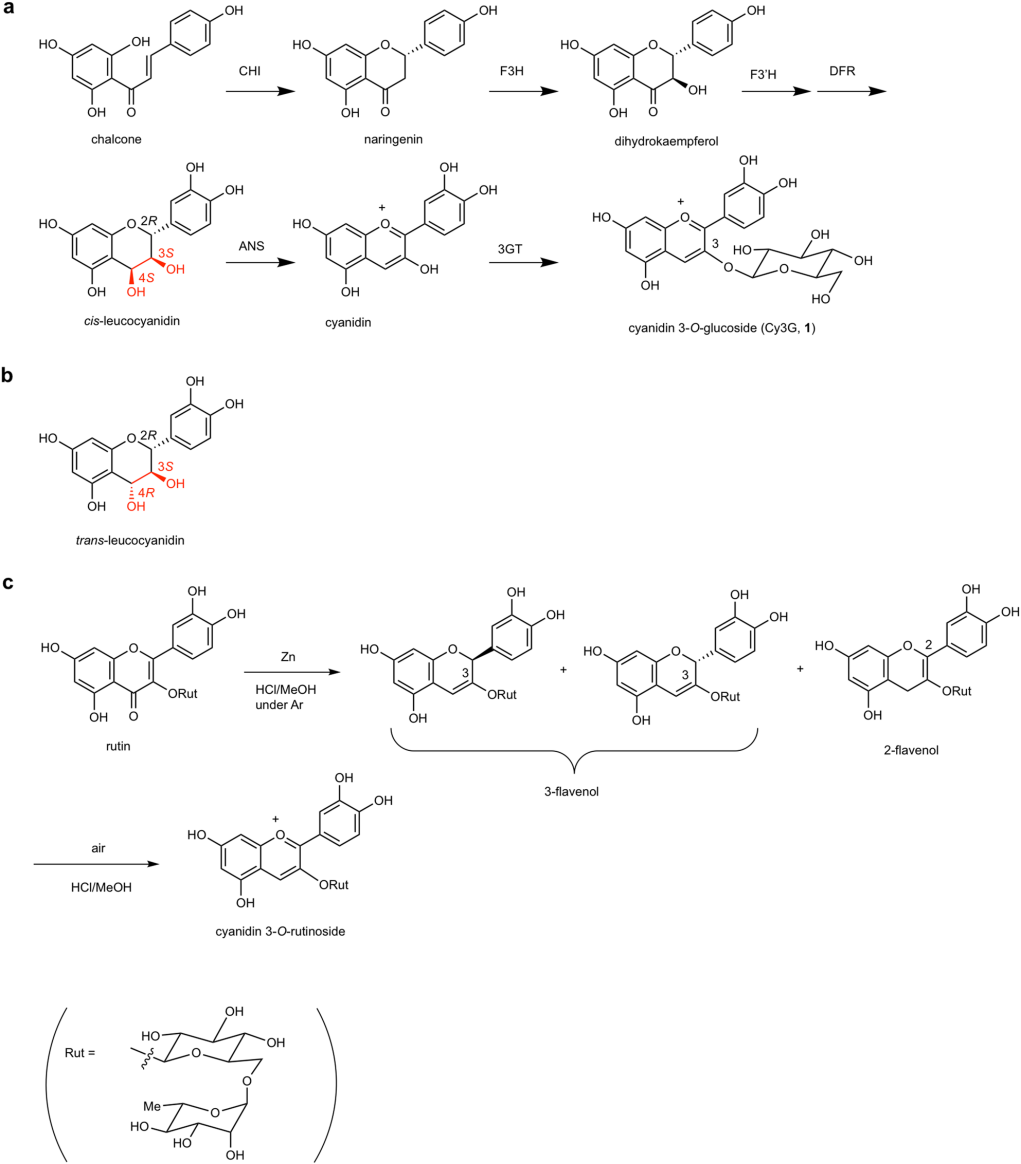
Late-steps in the biosynthetic pathway of cyanidin 3-*O*-glucoside (**1**). (**a**) Proposed pathway via* cis*-leucocyanidin (2*R*, 3*S*, 4*S*-leucocyanidin). (**b**) Structure of *trans*-leucocyanidin (2*R*, 3*S*, 4*R*-leucocyanidin). (**c**) Conversion of quercetin 3-*O*-glucoside to cyanidin 3-*O*-glucoside (**1**) by Clemmensen reduction followed by air oxidation via 2-flavonol 3-*O*-glucoside (2F3G, **2**) and 3-flavonol 3-*O*-glucosides.

The anthocyanin biosynthetic pathway diverges from the primary metabolites by the action of phenylalanine ammonia-lyase (PAL), which leads to the formation of naringenin upon cyclization. Several redox enzymes are then involved in its conversion to cyanidin, which constitutes the first colored compound in the pathway^15–20^. The last step in the conversion to cyanidin is catalyzed by anthocyanidin synthase (ANS), for which *cis*-leucocyanidin (2*R*, 3*S*, 4*S*-leucocyanidin) is presumed to be the substrate (Fig. 1a)^15,21–26^. However, *trans*-leucocyanidin (2*R*, 3*S*, 4*R*-leucocyanidin), is employed as the substrate in almost all reactions with recombinant enzymes^21–25^, as *cis*-leucocyanidin is unstable and commercially unavailable (Fig. 1b). Moreover, the detailed mechanism of the enzymatic reaction of ANS is still unclear^23,27^, and since cyanidin is not the major product in the enzymatic reaction of ANS, it has called into question the role of *cis*-leucocyanidin as the ANS substrate for transformation into cyanidin^26–31^. Furthermore, the enzymatic activity of ANS has been assayed by testing it for the production of Cy3G (**1**) using a mixture of ANS and anthocyanidin 3-*O*-glucosyltransferase (3GT), wherein the reaction sequence is unclear^23^. In addition, catechin was reported to form cyanidin upon treatment with ANS^27^. Overall, these results warrant a re-examination of the biosynthetic pathway of Cy3G (**1**) and the role of ANS.

Since we are interested in the reaction mechanism involved in the transformation of leucocyanidin to cyanidin, we undertook the chemical synthesis of Cy3G (**1**) and found that the oxidation of a *cis*-leucocyanidin derivative did not give Cy3G (**1**), but delivered a red-colored polymer^32^. To overcome this problem, we prepared a 3-flavenol derivative by 3,4-dehydration, which possessed the same oxidative level as *cis*-leucocyanidin and underwent facile air oxidation to furnish Cy3G (**1**) in a good yield^32^. Furthermore, we showed that the Clemmensen-type reduction of flavonol glycosides does not give the corresponding anthocyanin directly, but delivers initially a mixture of 2- and 3-flavenol compounds by reduction in the absence of air; these compounds are then oxidized by air in the second step to yield the corresponding anthocyanins (Fig. 1c)^33,34^. These findings therefore prompted us to study the mechanisms involved in the biosynthetic pathway of Cy3G (**1**) in the case of black soybean.

As reported previously, the green-colored seed matures to a black color over an approximately two-month period^35^, and this black color is known to be caused by the high quantities of cyanidin 3-*O*-glucoside (Cy3G, **1**) in the coating, i.e., approximately15 mg/g dried seed coat^15,36^. However, an accelerated change in the color of the beans from green to black was reported in normal pods upon accidental breakage of the shell. Around the same time as our synthetic studies, Fukami et al. reported the presence of a glucosylated 2-flavenol compound (i.e., 5,7,3ʹ,4ʹ-tetrahydroxyflav-2-en-3-ol 3-*O*-glucoside, 2F3G (**2**)) in the immature green-colored seed coat of black soybean^37^. They reported that the treatment of 2F3G (**2**) with hydrochloric acid affords Cy3G (**1**)^37^. Therefore, we wished to clarify this phenomenon in soybean tissue.

More specifically, we carried out a further examination, and found that immature green beans turned black within a day when the beans were removed from the pod and left under light and air, despite the fact that transformation to the black color takes approximately two months when the beans are inside the shell. We therefore determined the levels of Cy3G (**1**) and 2F3G (**2**) present in the immature seed coats and monitored their contents during the rapid color change. To achieve this objective, we synthesized 2F3G (**2**) from Cy3G (**1**) and determined their absolute purities using a quantitative NMR method (qNMR)^36–44^. Furthermore, non-enzymatic conversion from 2F3G (**2**) to Cy3G (**1**) in neutral aqueous solution was achieved. Thus, we herein propose that 2F3G (**2**) may be a biosynthetic precursor of cyanidin 3-*O*-glucoside (**1**) in the seed coat of black soybean.

## Results and discussion

### Rapid color change in the seed coat of immature black soybeans removed from the pod

**Figure 2:**
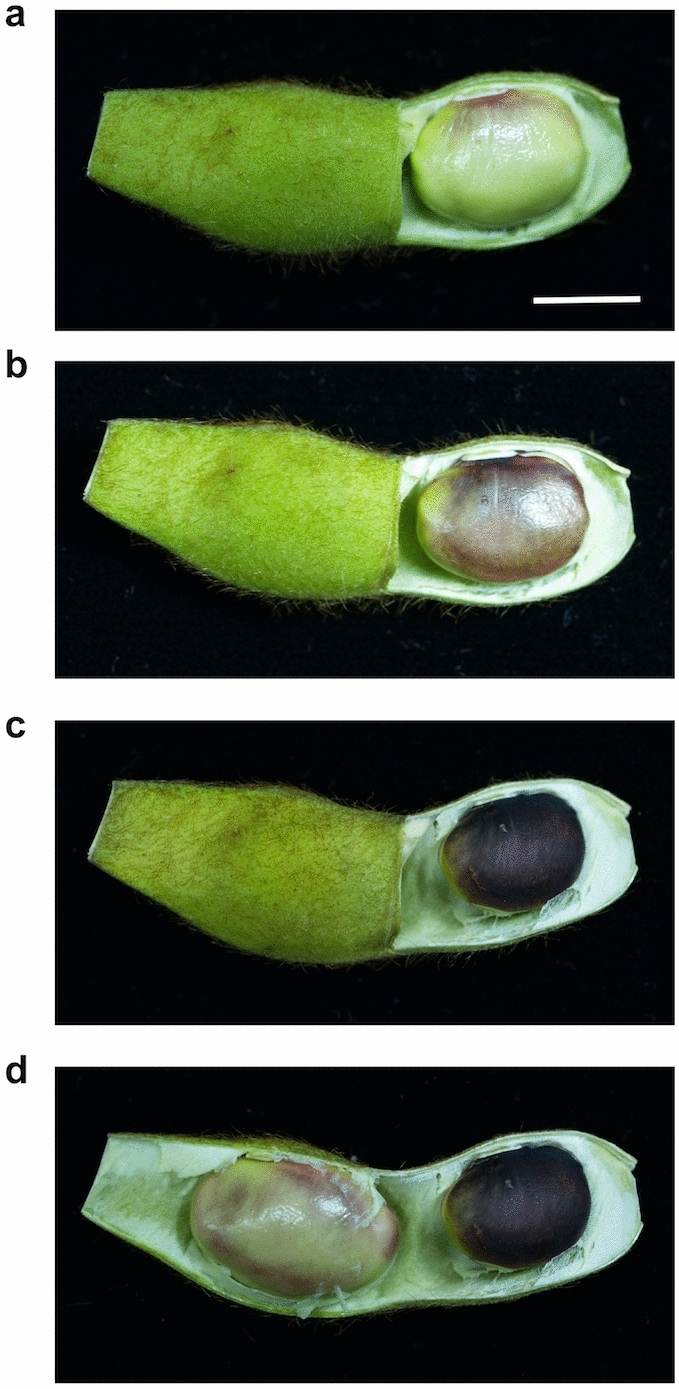
Rapid color change of the immature black soybean cv. Iwaikuro of stage 2. Half of the shell was removed and exposed to air under light conditions (20,000 l×) at 25 °C. (**a**) 0 h. (**b**) 10 h. (**c**) 20 h. (**d**) The remaining half was removed from the shell after 20 h. Scale bar: 1 cm.

To investigate the green-to-black color change phenomenon of the seed coat upon removal of the peels at the immature stage, and to determine whether formation of the black color is due to the presence of Cy3G (**1**), we cultivated *G. max* cv. Iwaikuro and harvested the immature green pods approximately 60 days after flowering. The shell of the right-hand half of the immature pod was removed and the partly peeled pod was incubated under light and air at 25 °C for 20 h (Fig. 2). The bean on the peeled side turned black gradually (Fig. 2b), and after 20 h of incubation, the black color was comparable to that of the mature beans (Fig. 2c). In contrast, the left-hand bean, which remained covered by the shell during the 20 h period, did not show any remarkable color change (Fig. 2d). The exposed immature beans that turned black upon incubation were then extracted using a mixture of 3% trifluoroacetic acid (TFA)-50% aq. acetonitrile (CH_3_CN) and analyzed by HPLC with photodiode array detection. The obtained chromatogram indicated the presence of one pigment, which was identified as Cy3G (**1**) by comparison with an authentic sample (Figure S1)^14^. The same phenomenon was observed when using other cultivars, i.e., *G. max,* cv. Murasaki-zukin (Figure S2), therefore indicating that this phenomenon may be not specific to cv. Iwaikuro, but could be universal. Since pigmentation of the seed coats of black soybeans in pods tends to proceed gradually^35^, this rapid accumulation of Cy3G (**1**) may indicate the presence of precursors for Cy3G (**1**) in the immature seed coat.

### Survey of 5,7,3ʹ,4ʹ-tetrahydroxyflav-2-en-3-ol 3-*O*-glucoside (2F3G, 2) in immature back soybean and its synthesis by the reduction of cyanidin 3-*O*-glucoside (1)

**Figure 3:**
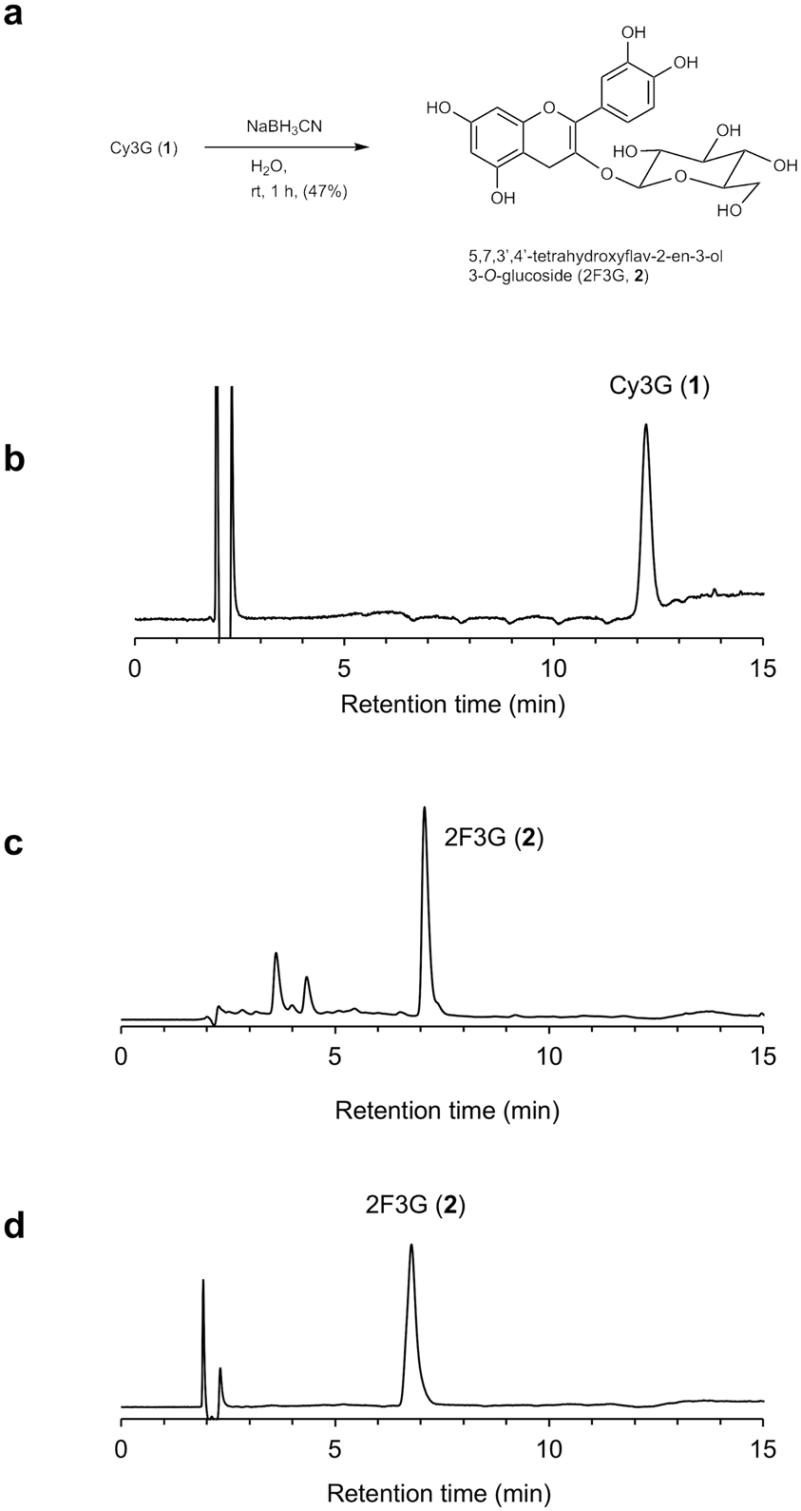
Synthesis of 5,7,3ʹ,4ʹ-tetrahydroxyflav-2-en-3-ol 3-*O*-glucoside (2F3G, **2**). (**a**) Scheme showing the reduction process. (**b**) HPLC chromatogram of the reaction mixture at the starting point. (**c**) HPLC chromatogram after 1 h. (**d**) HPLC chromatogram of purified **2**.

Initially, we attempted the isolation of 2F3G (**2**) from the immature black soybean cv. Murasaki-zukin according to the report by Fukami et al.^37^ but obtained less than 1 mg from 10 g of beans (see Supporting information, Figures S3–S6). As shown in Figure S7, the same compound, 2F3G (**2**), was also detected in black soybean cv. Iwaikuro. This indicates that regardless of the cultivar, the same compounds may be involved in the rapid biosynthesis of Cy3G (**1**) in immature black soybean seed coats. Since 2F3G (**2**) is labile to air and acids, and is easily converted to Cy3G (**1**), the isolation of 2F3G (**2**) from immature beans is highly challenging, and so we attempted the synthesis of 2F3G (**2**) for characterization and quantitative analysis. As we previously reported, the Clemmensen-type reduction of rutin under an argon atmosphere affords a mixture of 5,7,3ʹ,4ʹ-tetrahydroxyflav-2-en-3-ol 3-*O*-rutinoside and 5,7,3ʹ,4ʹ-tetrahydroxyflav-3-en-3-ol 3-*O*-rutinoside (Fig. 1c), however, the products are a mixture of 2- and 3-flavenol compounds^33,34^. On the other hand, the reduction of anthocyanins with NaBH_3_CN gives 5,7,3ʹ,4ʹ-tetrahydroxyflav-2-en-3-ol derivatives as the major products^33,34^. We therefore attempted the reduction of an aqueous solution of Cy3G (**1**) using 1 eq. of NaBH_3_CN (Fig. 3a). The HPLC chromatogram of the reaction mixture after 1 h indicated that the starting material was completely consumed, and three new peaks were detected. Upon comparison with the standard sample, the major peak was determined to be 2F3G (**2**), while the other two peaks were tentatively assigned to be diastereomers at the 2-position of 5,7,3ʹ,4ʹ-tetrahydroxyflav-3-en-3-ol 3-*O*-glucoside (Fig. 3b,c)^33,34^. Following evaporation and preparative HPLC purification of the resulting residue, pure 2F3G (**2**) was obtained in 47% yield (Fig. 3d), and its structure was confirmed by MS and NMR analyses (Figure S8 and S9, Table S3).

### Determination of the absolute purities of cyanidin 3-*O*-glucoside (1) and 5,7,3ʹ,4ʹ-tetrahydroxyflav-2-en-3-ol 3-*O*-glucoside (2) by qNMR measurements

Although HPLC is routinely used for quantitative analysis, such quantitation is feasible only when the necessary certified standards, which are limited, are available. For example, certified standards of anthocyanins are commercially unavailable despite their routine quantification in functional studies and in horticultural and food research. To address this issue, quantitative NMR (qNMR) methods have been developed to determine the absolute purities of organic compounds, particularly in the case of natural products, such as the anthocyanins^36–44^. Since Goda and his group reported that the purity of commercially available Cy3G (**1**) was < 90%^43^, we analyzed the prepared Cy3G (**1**) using the ^1^H-qNMR method. We also determined the purity of 2F3G (**2**), which was obtained from the conversion of Cy3G (**1**), and is highly unstable under acidic conditions, readily oxidizing to Cy3G (**1**).

The reported internal standard, 1,4-bis(trimethylsilyl) benzene-*d*_4_ was used^43^, and the method was slightly modified for instrument compatibility. The TFA-salt of Cy3G (**1**) was purified by re-precipitation and was obtained as a Cl-salt. Both Cy3G (**1**) and 2F3G (**2**) were dried under reduced pressure. The unfiltered sample solutions were then employed for subsequent ^1^H NMR measurements. The area of the signal corresponding to the H-4 proton was employed for the quantitation of Cy3G (**1**) (Figure S10), and the absolute purity of Cy3G (**1**) was determined in triplicate as 72.4 ± 3.9% (relative standard deviation (RSD), Table S4). Using the combined areas of the signals corresponding to H-2ʹ, 5ʹ, and 6ʹ, the purity of 2F3G (**2**) was determined to be 88.7 ± 0.7% (RSD, Figure S11, Table S5).

Since the obtained purity was rather low, the validity of this qNMR method was confirmed by measurement of our isolated Cy3G (**1**) using different instrument and an alternative external standard method. As shown in the Table S6 the purity of our isolated Cy3G (**1**) was the similar value. The purity value of the reagent Cy3G (**1**) quantified by qNMR method was also low (84.5%) compared with that obtained by HPLC analysis (> 98%). These data suggest that the low purities of Cy3G (**1**) and 2F3G (**2**) determined using the qNMR method may be due to the hygroscopic character of these compounds, or to some unknown basic problem in either the NMR measurements or during integration of the signal area. Further investigations are therefore required; however, for the purpose of this study, further quantification was carried out by HPLC using the obtained purity value indicated by qNMR.

### Contents of cyanidin 3-*O*-glucoside (1) and 5,7,3ʹ,4ʹ-tetrahydroxyflav-2-en-3-ol 3-*O*-glucoside (2) in the seed coats at different maturation stages

**Figure 4:**
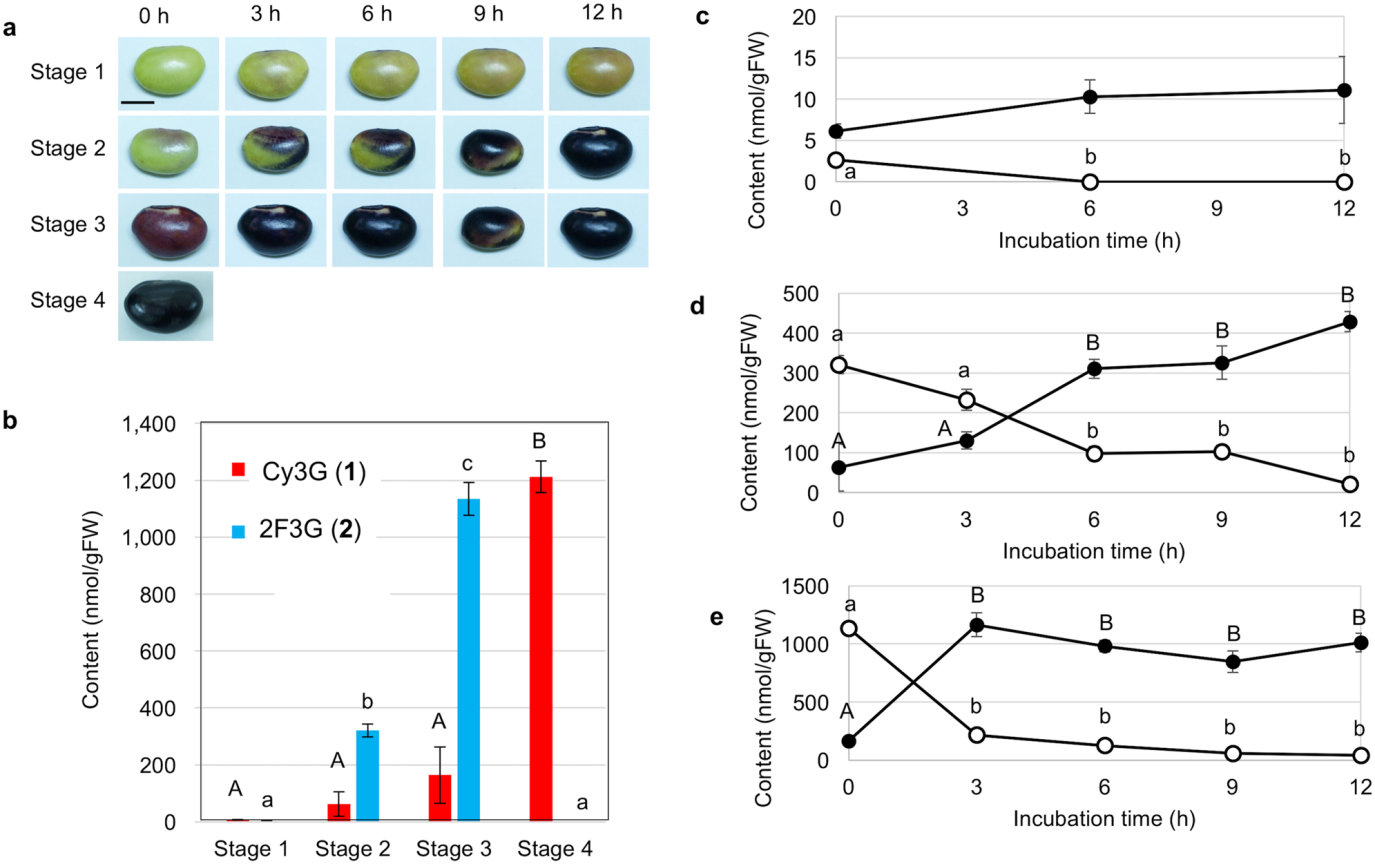
Contents and their changes of cyanidin 3-*O*-glucoside (Cy3G, **1**) and 5,7,3ʹ,4ʹ-tetrahydroxyflav-2-en-3-ol 3-*O*-glucoside (2F3G, **2**) in the beans at different maturation stages upon exposure to air under light conditions (20,000 l×) at 25 °C. (**a**) Color change of the seed coat in each developmental stage. (**b**) Contents of Cy3G (**1**) and 2F3G (**2**) in the seed coat of black soybean at each stage. Changes in the contents of Cy3G (**1**) and 2F3G (**2**) in the beans at stage 1 (**c**), the beans at stage 2 (**d**) and the beans at stage 3 (**e**). Each piece of data is a mean of three biological replicates displayed with the standard error of the mean. Significant differences (*P* < 0.05) observed between the data are indicated by different capital letters. Similarly, small letters indicate significant differences (*P* < 0.05). Scale bar: 1 cm.

We then analyzed the contents of Cy3G (**1**) and 2F3G (**2**) in the black soybean cv. Iwaikuro at different stages of maturation, as determined by the seed coat color (Fig. 4). More specifically, the green beans represented stage 1; the partially purple beans represented stage 2; the fully purple beans represented stage 3; and the fully black beans represented stage 4 (Fig. 4a). For each state, three beans were extracted with acidic and neutral solutions and were analyzed by HPLC. It was noted during the acidic extraction process that all of the 2F3G (**2**) present in the seed coat was converted to Cy3G (**1**), suggesting that the content of Cy3G (**1**) in the acidic extract indicates the combined amounts of Cy3G (**1**) and 2F3G (**2**). In contrast, only 2F3G (**2**) was present in the neutral extract. Thus, the true content of Cy3G (**1**) in the seed coat was calculated by subtraction of the content of 2F3G (**2**) from that of total Cy3G (**1**). The contents of Cy3G (**1**) and 2F3G (**2**) in the beans at each stage are shown in Fig. 4b. In stage 1, the contents of Cy3G (**1**) and 2F3G (**2**) are low. Upon maturation of the beans, however, the content of Cy3G (**1**) increased from 58 nmol/gFW (stage 2) to 166 nmol/gFW (stage 3) and 1200 nmol/gFW (stage 4), while the 2F3G (**2**) contents were approximately 320 nmol/gFW in stage 2, 1130 nmol/gFW in stage 3, and 0 nmol/gFW in stage 4. These results suggested that the biosynthesis of 2F3G (**2**) may begin around stage 2, while that of Cy3G (**1**) follows next.

### Changes of the contents of cyanidin 3-*O*-glucoside (1) and 5,7,3ʹ,4ʹ-tetrahydroxyflav-2-en-3-ol 3-*O*-glucoside (2) during incubation outside the pod

Subsequently, we analyzed the color changes of the beans and the changes in the Cy3G (**1**) and 2F3G (**2**) contents at each stage outside the pod. We removed the shells of the immature beans at stages 1–3, and exposed them to air under light conditions for 12 h. As shown in Fig. 4a, the beans at stage 1 did not show any remarkable color change, but the beans at stages 2 and 3 turned black following incubation. The contents of Cy3G (**1**) and 2F3G (**2**) in the various seed coats were then quantified (Fig. 4c–e). As mentioned above, the contents of both Cy3G (**1**) and 2F3G (**2**) were low, less than 10 nmol/gFW, at stage 1, with minimal changes in their content being observed; however, a decrease in the level of 2F3G (**2**) and an increase in that of Cy3G (**1**) were observed (Fig. 4c). At stage 2, the content of 2F3G (**2**) at 0 h was 305 nmol/gFW, and this decreased upon incubation to 220 (3 h), 100 (6 h), 100 (9 h), and 2 nmol/gFW (12 h) (Fig. 4d). In contrast with the decrease in 2F3G (**2**), the content of Cy3G (**1**) increased from 65 nmol/gFW (0 h) to 115 (3 h), 305 (6 h), 310 (9 h), and 415 nmol/gFW (12 h) (Fig. 4d). The total amount of Cy3G (**1**) and 2F3G (**2**) at each incubation time were 370, 405, 410, and 417 nmol/gFW, respectively, indicating that the total amount was constant. These results strongly indicate that the conversion of 2F3G (**2**) to Cy3G (**1**) occurs in the seed coat. At stage 3, a similar decrease in the level of 2F3G (**2**) correlating with an increase in that of Cy3G (**1**) was observed (Fig. 4e). The content of 2F3G (**2**) at 0 h was 1130 nmol/gFW and decreased rapidly as 216 nmol/gFW (3 h), 126 nmol/gFW (6 h), 59 nmol/gFW (9 h) and 40 nmol/gFW (12 h). The content of Cy3G (**1**) at 0 h was 164 nmol/gFW and increased to 1164 nmol/gFW at 3 h. However, the total amounts of Cy3G (**1**) and 2F3G (**2**) were almost constant value as 1400 nmol/gFW (0 h), 1450 nmol/gFW (3 h), 1106 nmol/gFW (6 h), 909 nmol/gFW (9 h) and 1051 nmol/gFW (12 h), respectively. These results strongly support that conversion from 2F3G (**2**) to Cy3G (**1**).

### Oxidation of 5,7,3ʹ,4ʹ-tetrahydroxyflav-2-en-3-ol 3-*O*-glucoside (2) to cyanidin 3-*O*-glucoside (1) by ferrous ions

Although it was previously reported that 2F3G (**2**) was converted to Cy3G (**1**) under acidic conditions, such as MeOH containing hydrochloric acid^37^, 2F3G (**2**) was found to be relatively stable in neutral aqueous solution. To determine whether an enzyme, such as ANS, may be involved in the conversion from 2F3G (**2**) to Cy3G (**1**) in the immature seed coat, we prepared a crude protein extract of the seed coat of an immature soy bean (stage 2) according to the report by Ohgami et al.^46^ Using the obtained protein extract, the conversion of 2F3G (**2**) to Cy3G (**1**) was examined using the conditions reported by Saito et al.^22^ More specifically, 2F3G (**2**) and the crude protein were added to the assay medium at pH 7 and reaction was monitored by HPLC (Figures S15a–c). After 48 h, 2F3G (**2**) remained in the mixture, although only a very small amount of Cy3G (**1**) was detected (Figure S15b). The presence of the crude protein did not affect the results, with the addition of heated protein giving similar results (Figure S15c). It was therefore concluded that the enzymatic reaction is likely not involved in the conversion reaction. In this context, we note that ANS was considered to be present in the cytosol, where the oxidation of *cis*-leucocyanidin to cyanidin was assumed to take place; however, if glycosylated substrate, 2F3G (**2**), is present in the vacuole, the pH should be < 7.0, i.e., around 5.0. We therefore attempted the assay at pH 5.0, and as shown in Figures S15e–f, after 48 h, the majority of 2F3G (**2**) had been consumed, and Cy3G (**1**) was found to be present (Figure S15e). The addition of heated protein resulted in a decrease in 2F3G (**2**) and an increase in Cy3G (**1**), although the reaction was slow compared to when the crude protein was employed (Figure S15f.).

**Figure 5:**
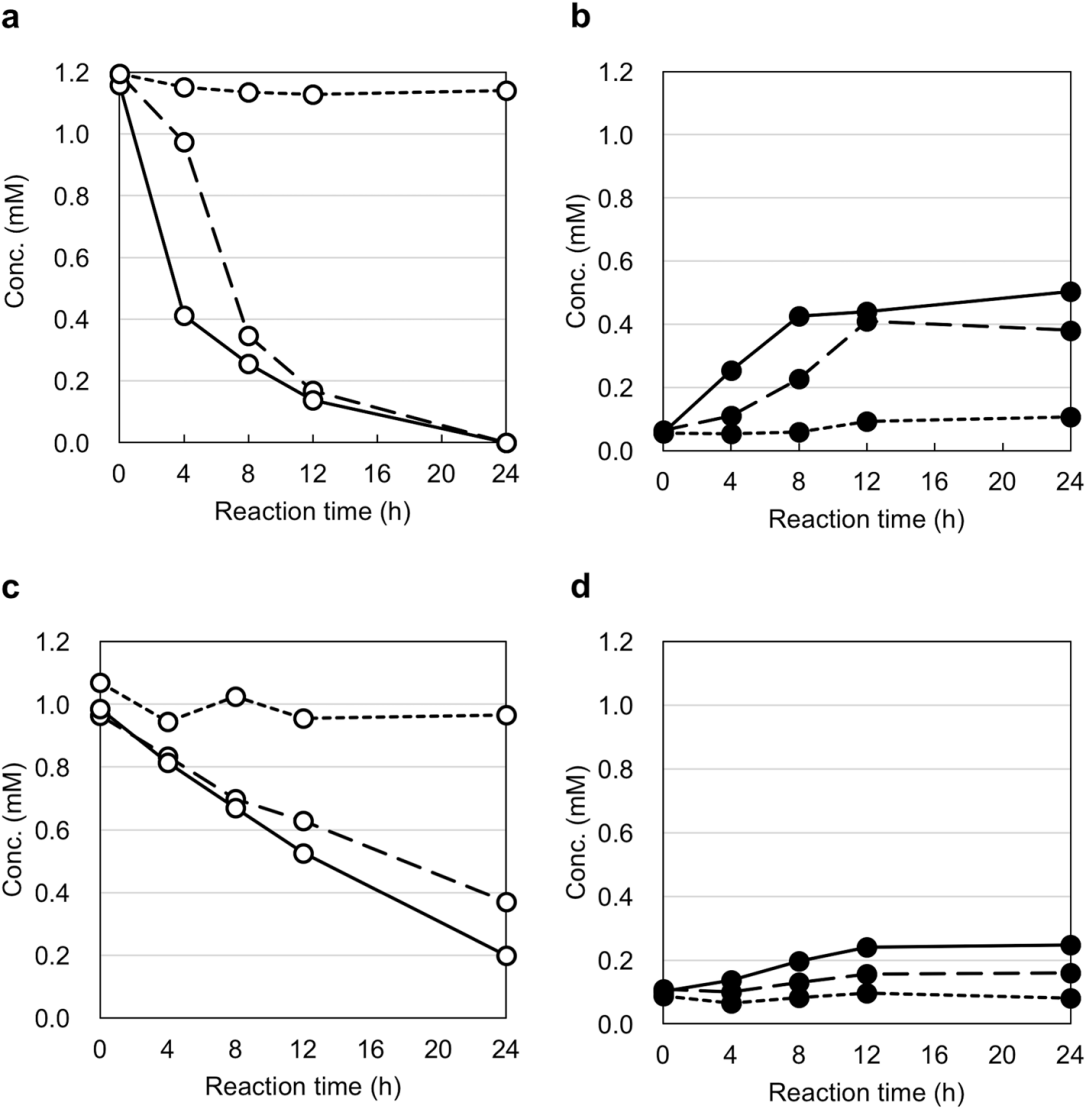
In vitro conversion of 5,7,3ʹ,4ʹ-tetrahydroxyflav-2-en-3-ol 3-*O*-glucoside (2F3G, **2**) to cyanidin 3-*O*-glucoside (Cy3G, **1**) in aqueous solutions with FeSO_4_ at 30 °C. Solid line: 0.4 mM FeSO_4_ + 4 mM sodium ascorbate, broken line: 0.4 mM FeSO_4_, dotted line: 4 mM sodium ascorbate. (**a**) Decrease in 2F3G (**2**) w/wo FeSO_4_ and sodium ascorbate at pH 5.0. (**b**) Increase in Cy3G (**1**) w/wo FeSO_4_ and sodium ascorbate at pH 5.0. (**c**) Decrease in 2F3G (**2**) w/wo FeSO_4_ and sodium ascorbate at pH 7.0. (**d**) Increase in Cy3G (**1**) w/wo FeSO_4_ and sodium ascorbate at pH 7.0.

To clarify the mechanism of this reaction, we prepared a simplified assay medium reducing the number of reagents one by one, and examined the conversion of 2F3G (**2**) to Cy3G (**1**). Interestingly, it was found that ferrous ions could catalyze the oxidation reaction at pH 5.0 (Fig. 5). In a buffered solution at pH 5.0, 2F3G (**2**) (1 mM) and 0.4 mM FeSO_4_ were dissolved and the resulting solution maintained at 30 °C. The contents of 2F3G (**2**) and Cy3G (**1**) in the reaction mixture were then quantified. At pH 5, both in the presence and absence of sodium ascorbate, the content of 2F3G (**2**) decreased, and this was accompanied by the corresponding increase in Cy3G (**1**) (Fig. 5a,b). In contrast, at pH 7, the content of Cy3G (**1**) was particularly low (Fig. 5c,d), and in the absence of ferrous ions, no conversion was observed at either pH 5 or 7. At both pH values, Cy3G (**1**) is stable over 24 h incubation, thereby indicating that the difference in the content of Cy3G (**1**) in the reaction mixture was not caused by any differences in stability. Furthermore, at both pH 5 and 7, the addition of sodium ascorbate slightly suppressed the decrease in 2F3G (**2**) and conversion to Cy3G (**1**). Chemically, the oxidative conversion of 2F3G (**2**) to Cy3G (**1**) may involve a radical reaction, and so the difference in reactivity between pH values of 5 and 7 may be due to differences in the degree of radical generation.

We herein reported our investigation into the color change taking place from green to purple-black in the immature seed coat of black soybean upon removal of the shell and exposure to light and air. We detected the presence of 5,7,3ʹ,4ʹ-tetrahydroxyflav-2-en-3-ol 3-*O*-glucoside (**2**) in the immature seed coat, and during the color change, a decrease in the content of 2F3G (**2**) was observed along with a simultaneous increase in the content of cyanidin 3-*O*-glucoside (**1**). These results strongly suggest the rapid conversion of 2F3G (**2**) to Cy3G (**1**) in the immature seed coat after removal of the shells. It was also observed in vitro that 2F3G (**2**) was oxidized to Cy3G (**1**) in the presence of air and ferrous ions at pH 5.0, and that the crude protein extract of the immature seed coat did not affect the conversion. These findings indicate that 2F3G (**2**) may exist in vacuoles, and that it is oxidized non-enzymatically by the ferrous ion acting as a catalyst, although the oxidizing agent involved in this transformation has yet to be clarified. Finally, based on our results, a new biosynthetic pathway to Cy3G (**1**) was proposed via a 3-*O*-glucosylation reaction prior to oxidation to the corresponding anthocyanidin.

## Methods

### General procedure and chemicals

All the procedures of instrumental analysis and chemicals used were written in supporting informaion.

### Plant material and treatment

Black soybeans (*Glycine max*) cv. Iwaikuro were donated by the Hokkaido Agricultural Research Center and were further cultivated at the Research Center Togo Field, Nagoya University, and Botanical Garden, Nagoya University Museum between 2015 and 2019. *G. max* cv. Murasaki-zukin was donated from Dr. Furuya of the Kyoto Prefectural Experimental Station. *G. max* cv. Hikariguro was purchased in a market. The beans were stored at 4 °C until required for use.

Qualitative color change observation was carried out by using an immature green colored pod of which half of the shell was peeled. Then, the pod was stood under a fluorescent light at 25 °C for 20 h.

For quantitative analysis, the immature black soybean pods were harvested approximately 60 days after flowering, and were maintained at 4 °C until required for use. The beans were then removed from the pod and separated into four stages according to the seed coat color: green, stage 1; partially purple, stage 2; purple, stage 3; and black, stage 4 (Fig. 4a). Each bean was placed in a glass bottle (10 mL) and incubated at 25 °C for 0, 3, 6, 9, or 12 h under light conditions (20,000 lx) in a plant incubator (MLR-350, SANYO Electric, Osaka, Japan). Each of the three treated beans was subjected to extraction with acidic and neutral solvents for the quantitative analyses of Cy3G (**1**) and 2F3G (**2**), respectively.

### Cyanidin 3-*O*-glucoside (Cy3G, 1)

Cyanidin 3-*O*-glucoside (**1**) was isolated as its trifluoroacetic acetate (TFA) salt from the seed coat of *G. max* cv. Hikariguro according to our previously reported procedure^14^^.^ The obtained Cy3G (**1**) was dissolved in 1% HCl-MeOH (10 mL), filtered (pore size: 0.45 µm), and diethyl ether (Et_2_O, 80 mL) was added to the filtrate. The resulting solution was maintained at room temperature and the obtained dark red precipitate was gathered as the chloride salt of Cy3G (**1**). The desired cyanidin 3-*O*-glucoside (**1**), obtained as a chloride salt, was dried over under reduced pressure.

### Synthesis of 5,7,3′,4′-tetrahydroxyflav-2-en-3-ol 3-*O*-glucoside (2F3G, 2)

The cyanidin 3-*O*-glucoside (**1**) TFA salt (105.0 mg, 0.187 mmol) was dissolved in H_2_O (3.0 mL) and NaBH_3_CN (12.4 mg, 0.197 mmol) was added to the solution. After stirring at room temperature for 1 h, the reaction mixture was filtered using a cartridge (pore size: 0.45 µm) and the filtrate was purified by preparative HPLC (RPAQUEOUS-AR-5 20 mm i.d*.* × 250 mm) with a 15% solution of CH_3_CN in water. The fraction containing 2F3G (**2**) was evaporated under reduced pressure to afford pure 2F3G (**2**) (39.2 mg, 47%) as a colorless mass.

### Quantitative NMR analysis (qNMR)

The absolute purities of Cy3G (**1**) and 2F3G (**2**) were obtained by ^1^H-qNMR analysis as reported by Uchiyama et al. with slight modifications^43^. An internal standard, 1,4-bis(trimethylsilyl) benzene-*d*_4_ (1,4-BTMSB-*d*_4_, 1 mg), and Cy3G (**1**) (5 mg) in 5% TFA *d*-CD_3_OD were transferred to an NMR tube without filtration, and the ^1^H-NMR spectrum was acquired using the parameters described in Table S2. The areas of the signals corresponding to the standard and to the H-4 proton in Cy3G (**1**) were calculated and used to obtain the absolute purity of Cy3G (**1**) (Figure S10, Table S4) based on Equation S1. For the measurement of 2F3G (**2**), the internal standard (0.5 mg) and 2F3G (**2**) (5 mg) were weighed precisely and dissolved in CD_3_OD. For calculation of the absolute purity of Cy3G (**1**), three protons on the B-ring (H-2ʹ, H-5ʹ, and H-6ʹ) were used (Figure S11, Table S5). Samples were prepared in triplicate and measured independently.

### Quantitative analyses of Cy3G (1) and 2F3G (2) by HPLC

For quantitative analysis, three immature beans at different stages and different exposure treatments were weighed and frozen using liq. N_2_. The extraction solvent (3% TFA-50% CH_3_CN aq, 3.0 mL/g FW) was added to the beans, and the sample was allowed to stand at room temperature in the dark for 24 h. The extract was then diluted five-fold with 3% TFA-H_2_O, subjected to cartridge filtration (pore size: 0.45 µm), and the filtrate analyzed by HPLC. For the quantitative analysis of 2F3G (**2**), three immature beans of different stages and different exposure treatments were weighed, frozen using liq. N_2_, and extracted with 50% aq. CH_3_CN (3.0 mL/g FW). The extract was then diluted five-fold with H_2_O, filtered through a cartridge (pore size: 0.45 µm), and analysis of the filtrate carried out by HPLC. For calibration, the dried 2F3G (**2**) was weighed precisely in triplicate, and the individual samples were diluted with MeOH. Each sample was analyzed by HPLC in triplicate (Table S7), and the obtained peak areas were used for construction of the calibration curve (Figure S14).

### Preparation of crude protein extract from immature seed coat and conversion of 2F3G (2) in vitro

From the immature seed coat of black soybean *G. max* cv. Iwaikuro, the crude protein extract was prepared and the conversion of 2F3G (**2**) to Cy3G (**1**) was carried out and monitored by HPLC (Figure S15). For detailed investigations, the composition of the assay medium was simplified, and the effect of the ferrous ion was examined. In an assay medium composed of sodium ascorbate (4 mM), FeSO_4_·7H_2_O (0.4 mM) in phosphate buffer (20 mM, pH 7.0), or acetate buffer (20 mM, pH 5.0), 2F3G (**2**) (1 mM) was added and the resulting mixture was incubated at 30 °C for 24 h. The reaction mixture was then diluted with 3% TFA aq. 5 times and analyzed by HPLC using the elution conditions outlined in Table S8.

### Statistical analysis

The quantitative analyses of Cy3G (**1**) and 2F3G (**2**) in the immature black soybean sample were performed in triplicate. The data were analyzed by one-way ANOVA with the post hoc Scheffe test. The significant differences are indicated in the figures using different characters as explained in the figure legends.

## Supplementary information

Supplementary information
