# Supplementary material for: 5,7,3ʹ,4ʹ-Tetrahydroxyflav-2-en-3-ol 3-O-glucoside, a new biosynthetic precursor of cyanidin 3-O-glucoside in the seed coat of black soybean, Glycine max
Source: Sci Rep. 2020 Oct 14;10:17184. doi: 10.1038/s41598-020-74098-6 (PMC7560818; doi:10.1038/s41598-020-74098-6)
Supplement: Supplementary file 1 — Supplementary information [file 41598_2020_74098_MOESM1_ESM.pdf]

Supporting Information

For

5,7,3′,4′-Tetrahydroxyflav-2-en-3-ol 3-*O*-glucoside, a new biosynthetic precursor of cyanidin 3-*O*-glucoside in seed coat of black soybean, *Glycine max*

Kumi Yoshida,[1]* Yada Teppabut,[1†] Reo Sawaguchi,[1†] Yuhsuke Nakane,[2] Emi Hayashi,[2] Kin-ichi Oyama,[3] Yuzo Nishizaki,[4] Yukihiro Goda[4] and Tadao Kondo[1]

[1]Graduate School of Informatics, [2]Graduate School of Information Science, Nagoya University, Chikusa, Nagoya 464-8601, Japan.
[3]Research Center for Materials Science, Nagoya University, Chikusa, Nagoya 464-8602, Japan.
[4]National Institute of Health Sciences, 3-25-26 Tonomachi, Kawasaki-ku, Kawasaki, Kanagawa 210-9501, Japan.

*To whom correspondence should be addressed.
E-mail: yoshidak@i.nagoya-u.ac.jp, Tel & Fax: +81-52-789-5638
[†]: These authors contribute equally.

Table of Contents

1. General P. 1
2. Chemical materials P. 1
3. Plant materials and treatment P. 2
4. Purification and preparation of Cl salt of cyanidin 3-*O*-glucoside (Cy3G, **1**) P. 2
5. HPLC analysis of the extract of blackened seed coat P. 3
6. Survey of 5,7,3',4'-tetrahydroxyflav-2-en-3-ol 3-*O*-glucoside (2F3G, **2**) in immature seed coat of black soybean P. 5
7. Synthesis of 5,7,3',4'-tetrahydroxyflav-2-en-3-ol 3-*O*-glucoside (2F3G, **2**) P. 11
8. Quantitative NMR analysis (qNMR)-1: method using internal standard P. 15
9. Quantitative NMR analysis (qNMR)-2: method using external standard P. 18
10. Quantitative analysis of Cy3G (**1**) by HPLC. P. 21
11. Quantitative analysis of 2F3G (**2**) by HPLC. P. 22
12. Preparation of crude protein extract from immature seed coat P. 23
13. *In vitro* conversion of 5,7,3',4'-tetrahydroxyflav-2-en-3-ol 3-*O*-glucoside (2F3G, **2**) to cyanidin 3-*O*-glucoside (Cy3G, **1**) by addition of crude extract P. 23
14. *In vitro* conversion of 5,7,3',4'-tetrahydroxyflav-2-en-3-ol 3-*O*-glucoside (2F3G, **2**) to cyanidin 3-*O*-glucoside (Cy3G, **1**) by Ferrous ion without crude protein P. 24
15. Statistical Analysis P. 25
16. References P. 25

1. General

Melting points (mp) were determined on a Yanaco MP-3 instrument (Anatec Yanako, Kyoto, Japan) and are uncorrected. Optical rotations were recorded on a JASCO P-1010-GT polarimeter (Jasco Co. Hachioji, Japan). Infrared (IR) spectra were recorded on a JASCO FT/IR 6100 spectrometer (Jasco, Hachioji, Japan). The UV-Vis absorption spectrum was recorded with a JASCO V-560 spectrometer (Jasco, Hachioji, Japan). ESI-TOF-MS and HR-MS were recorded on a Bruker micrOTOF-QII (ESI) spectrometer (Bruker, Billerica, MA, USA). LC-MS analysis was carried out with an Agilent 1200 Series instrument (Agilent Technologies, Santa Clara, CA, USA). ^{1}H NMR spectra were recorded (5.0 mm i.d. tube) on a Bruker AVANCE III HD600 spectrometer (^{1}H: 600 MHz, ^{13}C: 150 MHz, Bruker, Billerica, MA, USA) and a JEOL ECA-500 spectrometer (^{1}H: 500 MHz, ^{13}C: 125 MHz, JEOL, Akishima, Japan). Chemical shifts for ^{1}H-NMR are reported on a scale relative to tetramethylsilane (TMS) (0.00 ppm) or CD_2HOD (3.31 ppm) in deuterated methanol. Chemical shifts for ^{13}C NMR were reported in the scale relative to the NMR solvent ($CDCl_3$: δ 77.0 ppm, CD_3OD: δ 49.0 ppm) as an internal reference. Analytical HPLC was performed using a JASCO HPLC system (Jasco, Hachioji, Japan) comprising two PU-1585 pumps, an HG-1580-32 mixer, a DG-1580-53 degasser, an MD-1515 detector, and a CO-1565 column oven. The system was controlled using a ChromNAV ver 2 application.[1] Reversed-phase columns (Develosil RPAQUEOUS-AR-3, 2.0 mm i.d. × 150 mm, Nomura Chemical, Seto, Japan) were employed, and elution was carried out using a linear gradient elution from 10% to 90% aq. CH_3CN solution containing 0.5% TFA at 40 °C. Preparative HPLC was carried out using a JASCO preparative HPLC system (Jasco, Hachioji, Japan) comprising a 880-PR pump, a UV-970 detector, a RC-250 recorder and a thermostatic oven (Jr-80, TAITEC, Koshigaya, Japan).[43] The Develosil RPAQUEOUS-AR-5 columns (4.6 mm i.d. × 250 mm, 8mm i.d. × 250 mm and 20 mm i.d. × 250 mm, Nomura Chemical, Seto, Japan) were eluted with a stepwise gradient from 10% to 50% aq. CH_3CN with and without 0.5% TFA at 40 °C.

2. Chemical materials

The inner standard, 1,4-bis (trimethylsilyl) benzene-d_4 (1,4-BTMSB-d_4), required for the quantitative NMR (qNMR) measurements was purchased from Fujifilm Wako Pure Chemical Corporation (Osaka, Japan). The external standard, deuterated 3-(trimethylsilyl)-1-propanesulfonic acid sodium salt, 2,2-Dimethyl-2-silapentane-5-sulfonate-d_6 sodium salt (DSS-d_6) was purchased from Fujifilm Wako Pure Chemical Corporation (Osaka, Japan). Reagent cyanidin 3-*O*-glucoside (Cy3G, **1**) was purchased from Tokiwa Phytochemical

(Chiba, Japan). 3-[(3-Cholamidopropyl)dimethylammonio]propanesulfonate was purchased from DOJINDO LABORATORIES (Kumamoto, Japan). DOWEX lx2 50-100 was purchased from The Dow Chemical Company (Midland, USA). All other commercially available reagents and solvents were purchased from Fujifilm Wako Pure Chemical Corporation, NACALAI TESQUE, INC (Kyoto, Japan), Tokyo Chemical Industry Co., Ltd (Tokyo, Japan) Sigma-Aldrich Japan (Tokyo, Japan).

3. Plant materials and treatment

Black soybeans (*Glycine max*) cv. Iwaikuro were donated by the Hokkaido Agricultural Research Center and were further cultivated at the Research Center Togo Field, Nagoya University, and Botanical Garden, Nagoya University Museum. *G. max* cv. Murasaki-zukin was donated by the Kyoto Prefectural Experimental Station. *G. max* cv. Hikariguro was purchased in a market. For analysis, immature black soybean pods were harvested approximately 60 days after flowering and maintained at 4 °C until required for use. Qualitative color change observation was carried out by using an immature green colored pod of which half of the shell was peeled. Then, the pod was stood under a fluorescent light at 25 °C for 20 h. For quantitative analyse, the beans were removed from the pod and separated into four stages according to the seed coat color: green, stage 1; partially purple, stage 2; purple, stage 3; and black, stage 4 (Fig. 4a). Each bean was placed in a glass bottle (10 mL) and incubated at 23 °C for 0, 3, 6, 9, and 12 h under light conditions (20,000 lx) in a plant incubator (MLR-350, SANYO Electric, Osaka, Japan). Each of the three treated beans was subjected to extraction with acidic and neutral solvents for the quantitative analyses of **1** and **2**, respectively.

4. Purification and preparation of Cl salt of cyanidin 3-*O*-glucoside (Cy3G, 1)

Cyanidin 3-*O*-glucoside (**1**) was isolated as a trifluoroacetic acetate (TFA) salt from the seed coat of *G. max* cv. Hikariguro according to our previously reported procedure with a light modification.[2] In detail, to 10 Kg of dried and matured black soybean *G. max* cv. Hikariguro 22 L of H_2O was added and stood in dark at room temperatre for 10 h, then the water-absorbed beans were frozen at –30 °C for overnight. The frozen beans were extracted with 0.7% HCl aq. (5 L) and the obtained extract was evaporated under reduced pressure to 1 L. To the concentrated extract 3 L of H_2O was added, then, the diluted extract was absorbed to Amberlithe XAD-7 gel (ORGANO, Tokyo, Japan, 2 L). The gel was washed with 0.2% TFA aq. (5 L), then, pigments was eluted with 0.2% TFA-50% CH_3CN aq. followed by dried up to

be obtained crude pigment (10 g). The crud pigment was purified by using Amberlithe XAD-7 column chromatography by elution with aq. MeOH (10%–50%) solution containing 0.2% TFA, repeatedly to give Cy3G (**1**, 416 mg, relative purity by HPLC: 98%). The obtained **1** (TFA-salt, 49.5 mg, 88 μmol) was dissolved in 1% HCl-MeOH (10 mL), and then filtered (pore size: 0.45 μm). Diethyl ether (Et_2O, 80 mL) was added to the filtrate, and the solution was maintained at room temperature. The resulting dark red precipitate was gathered by centrifugation and washed twice with Et_2O. The desired cyanidin 3-*O*-glucoside (**1**), obtained as a chloride salt, was dried over under reduced pressure. mp: 240–245 °C decomposed; UV/Vis (0.1% HCl-MeOH) λ_{max} nm (ε): 529 (30,400), 282 (17,233); IR (KBr) 3346, 1638, 1575, 1331, 1281, 1241, 1200, 1163, 1070 cm^{-1}; HRMS (ESI) calcd for $C_{21}H_{21}O_{11}$ $[M]^+$ 449.1078, found 449.1081; Anal. calcd for $C_{21}H_{21}ClO_{11}$•$(H_2O)_{1.5}$•$(CH_3CN)_{0.3}$•$(Et_2O)_{0.1}$: C, 49.71; H, 4.91; N, 0.79. Found: C, 49.75; H, 4.67; N, 0.71.

5. HPLC analysis of the extract of blackened seed coat

Immature black soybean (*G. max* cv. Iwaikuro) was exposed to air in light condition for 10 h. The blackened beans were frozen with liq. N_2 and extracted with approximately 10 volumes of a solution of 3% TFA-50% aq. CH_3CN in dark at room temperature for 5 h. The extract was filtered using a cartridge filter (pore size: 0.45 μm), and the filtrate was analyzed by HPLC by a linear gradient elution as shown in Table 1. Fig. S1a shows the chromatogram of the extract with 3D spectrum of the peak 1 (Fig. S1b). By comparison with the authentic sample (Fig. S1c), the peak 1 was identified to be Cy3G (**1**).

Table S1. HPLC condition for analysis of 1.

Column	RPAQUEOUS-AR-3, 2.0 mm i.d.×150 mm
Mobile phase	A: 0.5%TFA-10%CH_3CN aq.
	B: 0.5%TFA-90%CH_3CN aq.
Gradient condition	Linier gradient elution
0 min	A: 100%
5 min	A: 100%
17 min	A: 94%
22 min	A: 94%
Flow rate	0.2 mL/min
Detection	Photodiode array 200-650 nm
Temperature	40 °C

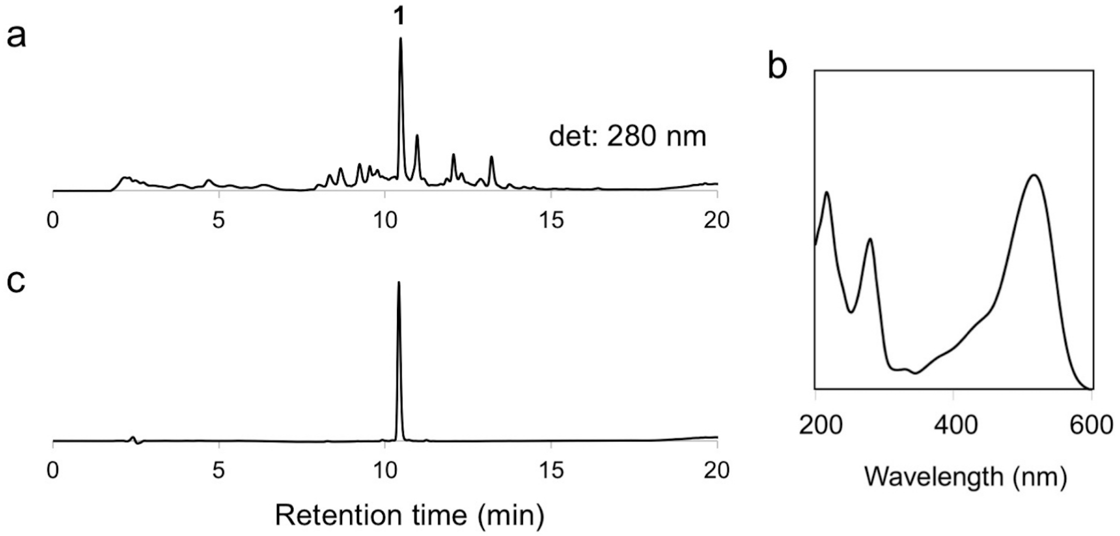

Figure S1. HPLC chromatogram of the extract of black-colored immature seed coat obtained exposed to light and air. a, HPLC chromatogram of the extract. **b**, UV-Vis spectrum of peak 1 in A obtained by photodiode array detection. **c**, HPLC chromatogram of standard cyanidin 3-*O*-glucoside (**1**).

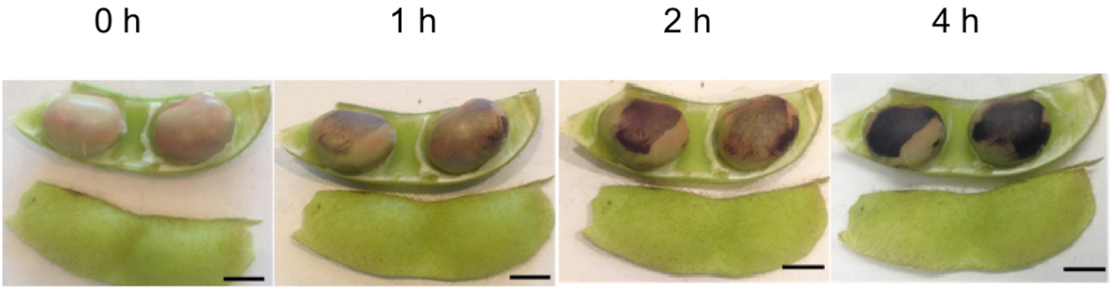

Figure S2. Rapid seed coat color change of immature black soybean taken out of pod. A one side-peel of immature black soybean (*G. max* cv. Murasaki-zukin) was removed and exposed to air in light condition for 4 h. Scale bar: 1 cm.

6. Survey of 5,7,3',4'-tetrahydroxyflav-2-en-3-ol 3-*O*-glucoside (2F3G, 2) in immature seed coat of black soybean

Five grams of immature beans of cv. Murasaki-zukin (stage 2) were extracted with 50% aq. CH_3CN for 5 h and the extract was analyzed by HPLC with photodiode array detection using the analytical condition of Table S2 (Fig. S3b). The spectrum of peak 2 showed the λmax around 279 nm (Fig. S3c) and the spectral shape was similar to that of 5,7,3',4'-tetrahydroxyflav-2-en-3-ol 3-*O*-rutinoside.[3,4] The peak 2 in the LC-MS analysis of the extract indicated a molecular ion at *m/z* = 451 (Fig. S3d), which suggests that peak 2 might be 5,7,3',4'-tetrahydroxyflav-2-en-3-ol 3-*O*-glucoside (2F3G, **2**, Fig. S3a).[5]

Frozen black soybeans cv. Murasaki-zukin ((stage 2, 10 g) were extracted with 50% aq. CH_3CN for 5 h, and the extract was evaporated under reduced pressure. The resulting mass (367 mg) was dissolved in H_2O and purified using RPAQUEOUS-AR-5 (4.6 mm i.d. × 250 mm) by elution with a gradient from 10 to 50% aq. CH_3CN. The fraction containing **2** was dried under reduced pressure to afford 2F3G (**2**) as a colorless mass (0.8 mg).

The HR-ESI-MS of which in the negative mode indicated the molecular ion peak at *m/z* 449.1086 (calcd. for $C_{21}H_{21}O_{11}$: 449.1089, Figure S4). Using various 1D and 2D NMR, the structure was identified to be 5,7,3',4'-tetrahydroxyflav-2-en-3-ol 3-*O*-glucoside (Fig. S5, S6, Table S3).

Five grams of immature beans of cv. Iwaikuro were extracted and analyzed by HPLC using the same procedure. **2** was detected as the same as that of Murasaki-zukin (Fig. S7).

Table S2. HPLC condition for analysis of 2.

Column	RPAQUEOUS-AR-3, 2.0 mm i.d.×150 mm
Mobile phase	A: 0.5%TFA-10%CH_3CN aq.
	B: 0.5%TFA-90%CH_3CN aq.
Gradient condition	Linier gradient elution
0 min	A: 100%
5 min	A: 94%
15 min	A: 94%
20 min	A: 70%
Flow rate	0.2 mL/min
Detection	Photodiode array 200-650 nm
Temperature	40 °C

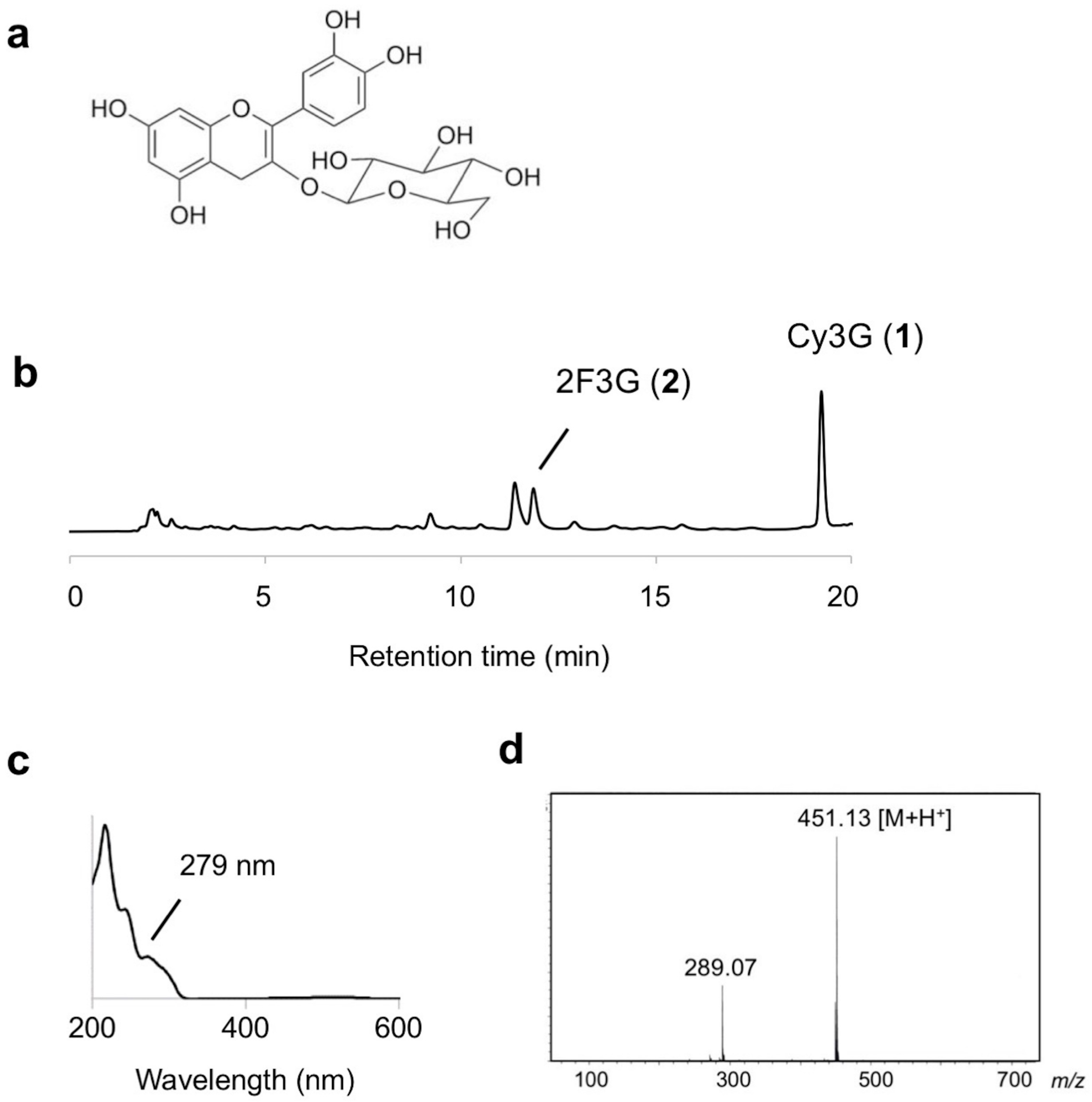

Figure S3. Analysis of immature black soybean extract (*G. max* cv. Murasaki-zukin). a, Structure of 5,7,3’,4’-tetrahydroxyflav-2-en-3-ol 3-*O*-glucoside (2F3G, **2**). **b**, HPLC chromatogram of the extract detected at 280 nm. **c**, Spectrum of **2** by photo-diode array detection. **d**, Mass spectrum obtained by LC-MS of **2**.

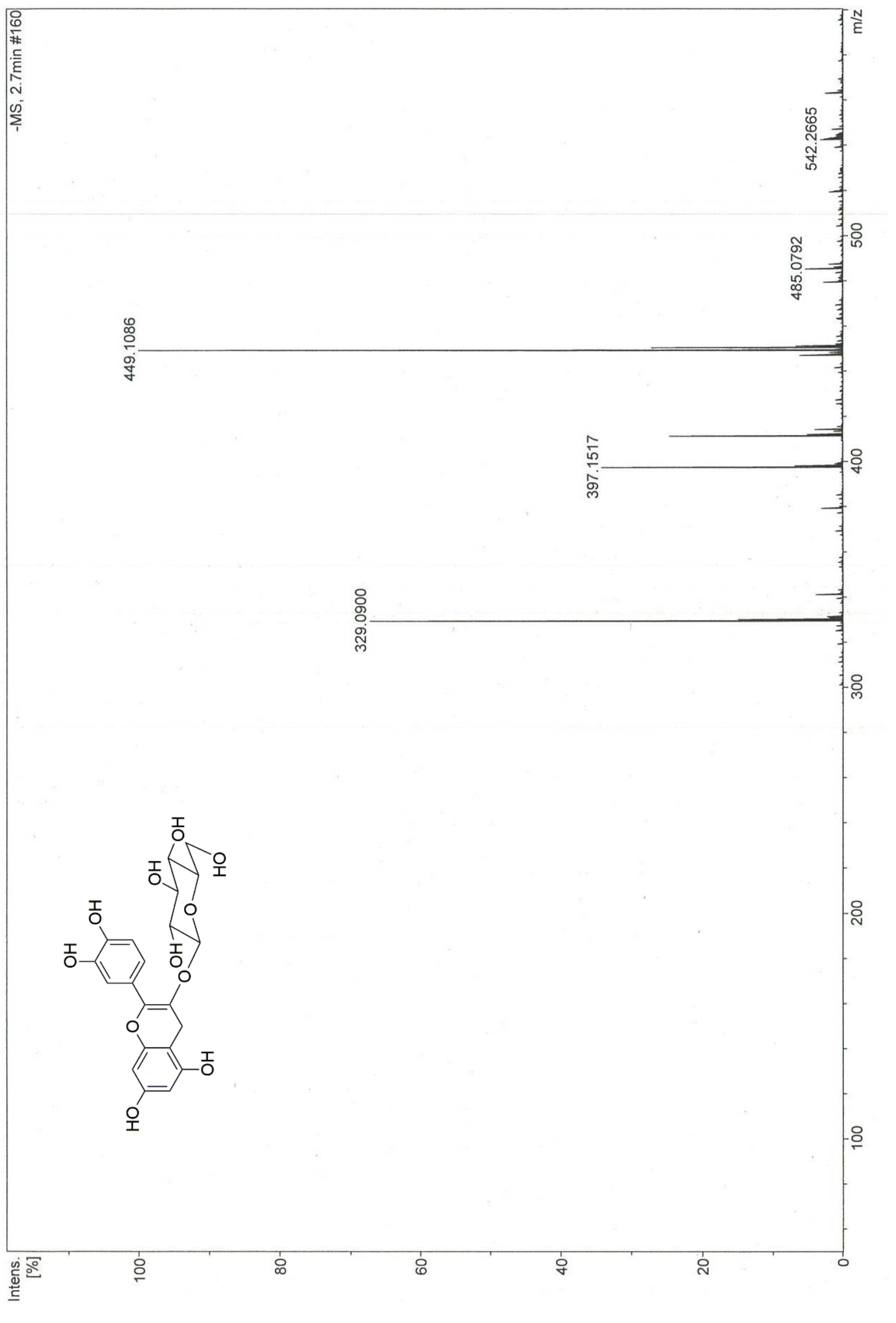

Figure S4. HR-ESI-MS of isolated 2.

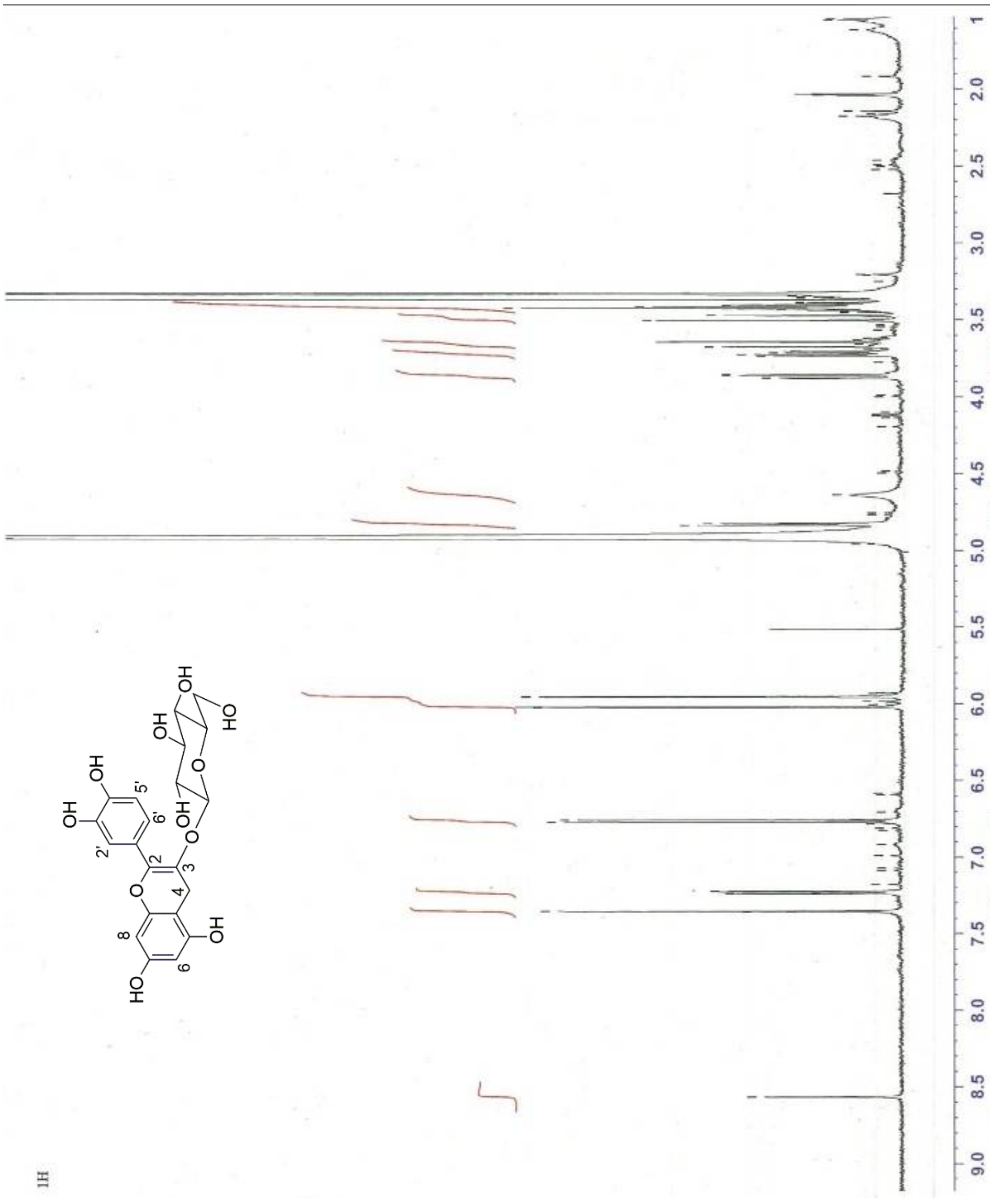

Figure S5. ^{1}H NMR spectrum of isolated 2. (^{1}H: 600 MHz, CD_3OD, 25 °C).

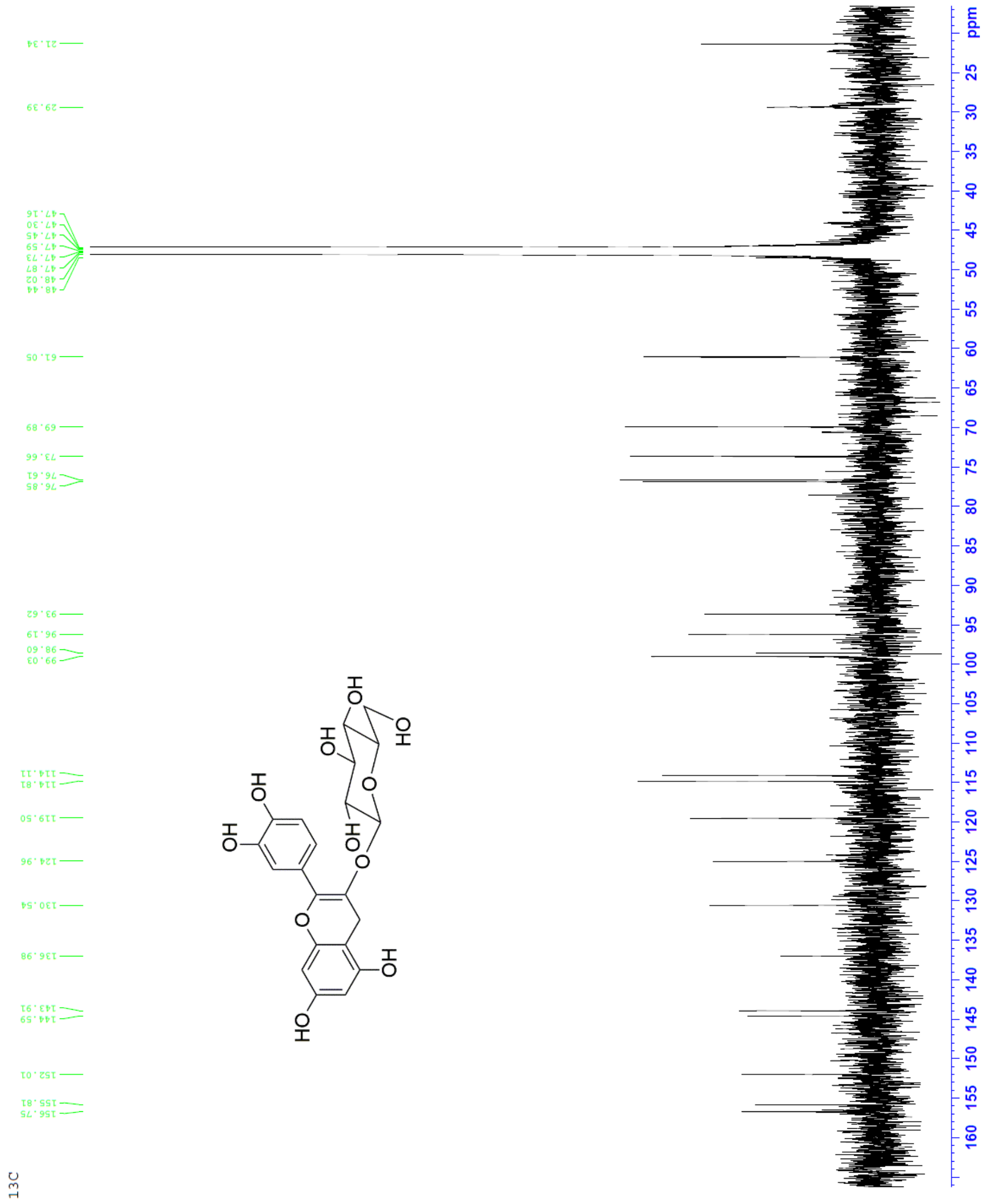

Figure S6. ^{13}**C NMR spectrum of isolated 2. (**^{13}**C: 150 MHz, CD_3OD, 25 °C).**

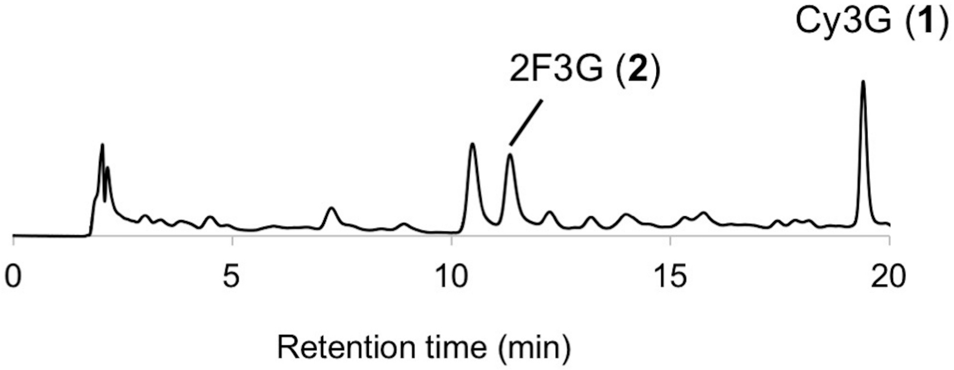

Figure S7. HPLC chromatogram of immature black soybean extract (*G. max* cv. Iwaikuro) detected at 280 nm.

7. Synthesis of 5,7,3',4'-tetrahydroxyflav-2-en-3-ol 3-*O*-glucoside (2F3G, 2)

The cyanidin 3-*O*-glucoside (**1**) TFA salt (105.0 mg, 0.187 mmol) was dissolved in H_2O (3.0 mL) and $NaBH_3CN$ (12.4 mg, 0.197 mmol) was added to the solution. After stirring at room temperature for 1 h, the reaction mixture was filtered using a cartridge (pore size: 0.45 μm) and the filtrate was purified by preparative HPLC (RPAQUEOUS-AR-5 20 mm i.d. × 250 mm) with a 15% solution of CH_3CN in water. The fraction containing **2** was evaporated under reduced pressure to afford pure **2** (39.2 mg, 47%) as a colorless mass. For qNMR, **2** was dried over under reduced pressure. mp: 200–210 °C decomposed; UV (MeOH) λ_{max} nm (ε): 298 (5176), 273 (7092), 246 (14892); $[\alpha]_D^{22} = -10.5$ (c 0.1, CH_3OH); IR (KBr) 3442, 3267, 2915, 1615, 1514, 1475, 1335, 1294, 1185, 1147, 1042, 795 cm^{-1}; HRMS (ESI) calcd for $C_{21}H_{21}O_{11}$ $[M–H]^-$ 449.1089, found 449.1086; Anal. calcd for $C_{21}H_{22}O_{11}$• (H_2O): C, 53.85; H, 5.16; N, 0.00. Found: C, 53.66; H, 4.94 N, 0.00.

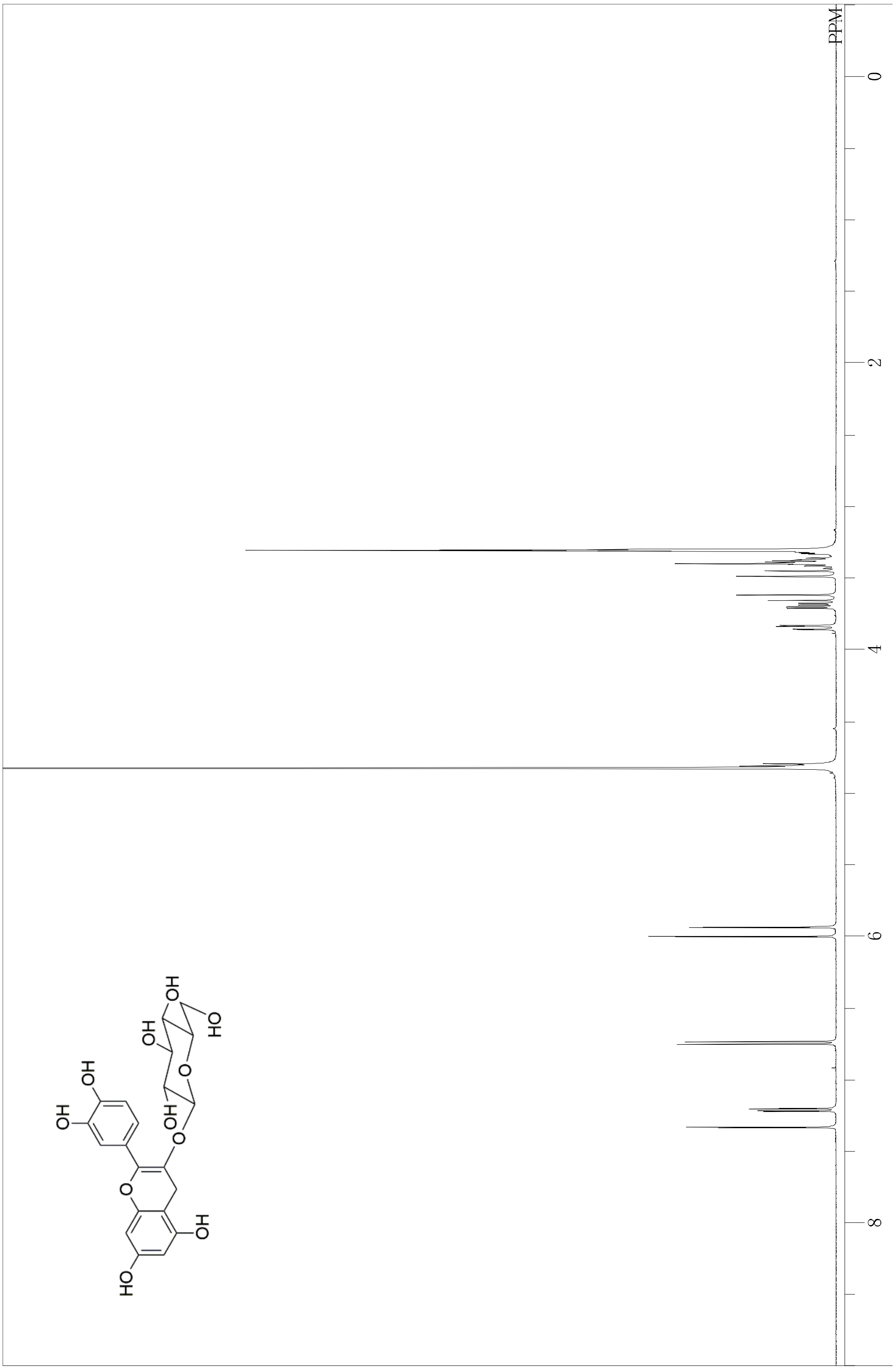

Figure S8. ^{1}H NMR spectrum of synthetic 2. (^{1}H: 500 MHz, CD_3OD, 25 °C).

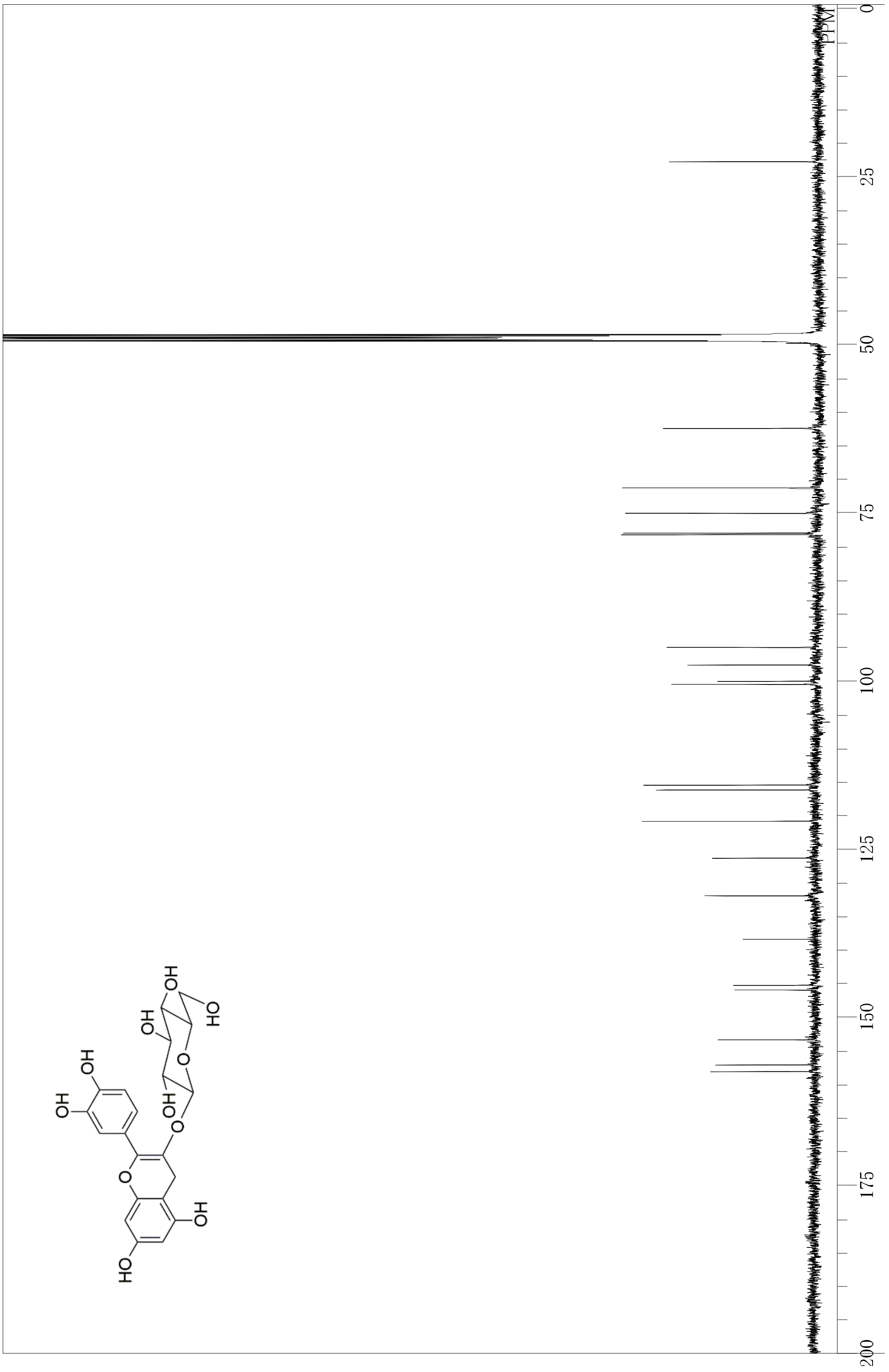

Figure S9. ^{13}C NMR spectrum of synthetic 2. (^{13}C: 125 MHz, CD_3OD, 25 °C).

Table S3. Assignment of ^{1}H and ^{13}C NMR of 2.
(^{1}H: 500 MHz, ^{13}C: 125 MHz CD_3OD, 25 °C)

	^{1}H			^{13}C
	δ (ppm)	multiplicity	*J* (Hz)	δ (ppm)
2				138.5
3				132.0
4a	3.64	d	19.0	22.8
4b	3.47	d	19.0	
5				157.2
6	6.01	d	2.0	97.7
7				158.1
8	5.94	d	2.0	95.1
9				153.4
10				100.1
1’				126.4
2'	7.34	d	1.5	116.3
3’				145.3
4’				146.0
5’	6.75	d	8.0	115.5
6’	7.22	dd	8.0, 21.5	120.9
G1	4.81	d	7.0	100.5
G2	3.39	m		75.1
G3	3.41	m		78.0
G4	3.38	m		71.3
G5	3.33	ddd	9.0, 5.5, 2.0	78.3
G6a	3.85	dd	12.0, 2.0	62.5
G6b	3.70	dd	12.0, 5.5	

8. Quantitative NMR analysis (qNMR)-1: method using internal standard

The absolute purities of **1** and **2** were obtained by ^{1}H-qNMR analysis as reported by Uchiyama et al. with slight modifications.[5] More specifically, a solution of the precisely weighed internal standard, 1,4-bis(trimethylsilyl) benzene-d_4 (1,4-BTMSB-d_4, 1 mg), and **1** (5 mg) in 5% TFA d-CD_3OD (0.6 mL), and was transferred to an NMR tube (5 mm i.d.) without filtration. All experiments for EC-qNMR analysis were performed operating with JEOL ECA-500 at 500.15592 MHz equipped with a 5-mm 50TH5AT/F62 probe and probe temperature was regulated at 25 ºC (298 K). The NMR operating software used was JEOL Delta v4.3.6. After the sample loading, shimming was done, then the probe was automatically tuned and matched. Before measurement the values 90PW was determined to be 12.5 μsec. For EC-qNMR of samples, acquisition of the ^{1}H spectrum was performed by using “single_pulse.ex2” pulse sequence. The NS was set to 8 and the dummy scans (ds) was set to 2. The relaxation delay (D_1) was set to 60 sec for maintaining the pulse repetition time (T_r) [AQ + D_1], is at least ten times the longest T_1 for the signals interested. The AQ was unified to 5.23 sec by setting the number of data points to 65,536 at a sufficient spectral width of 10,003 Hz. The FID was zero filled to 65,536 points (1 times) with trapesoid3 window functions. Then the phase of the spectrum was adjusted manually. The processed spectrum was saved separately from the original FID. The Signal(s) were automatically integrated. The areas of the signals corresponding to the standard and to the H-4 proton in **1** were calculated and used to obtain the absolute purity of **1** (Fig. S10, Table S4) based on Equation S1. where the subscripts denote the substances, analyte (A) and calibrant (C); S: absolute integral value, H: number of protons, m: weight, M: molecular weight. For the measurement of **2**, the internal standard (0.5 mg) and **2** (5 mg) were weighed precisely and dissolved in CD_3OD. For calculation of the absolute purity of **1**, three protons on the B-ring (H-2′, H-5′, and H-6′) were used (Fig. S11, Table S5). Samples were prepared in triplicate and measured independently.

Equation S1

$$P_A = S_A/S_C \times H_C/H_A \times m_C/m_A \times M_C/M_A$$

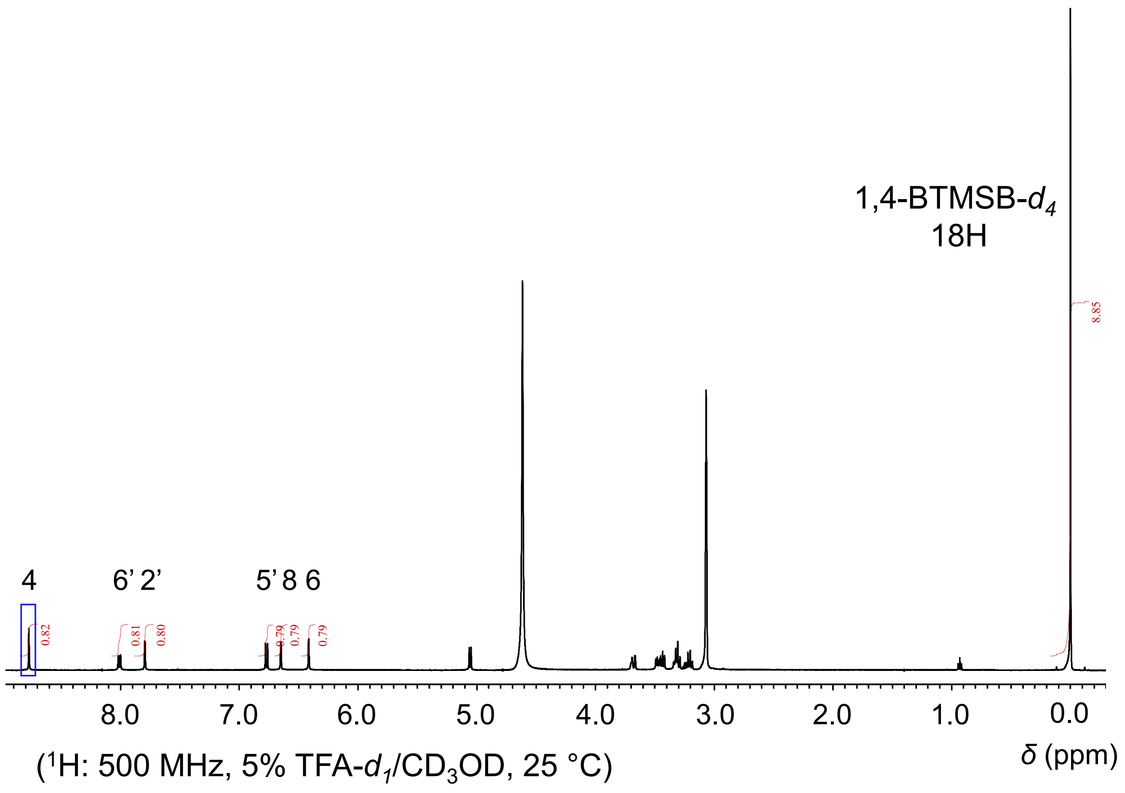

Figure S10. ^{1}H NMR spectrum of 1 with internal standard.

Table S4. Absolute purity of 1.

Entry	Purity (%)	Average (%)	RSD (%)
1	72.8		
2	68.8	72.4	3.9
3	75.6		

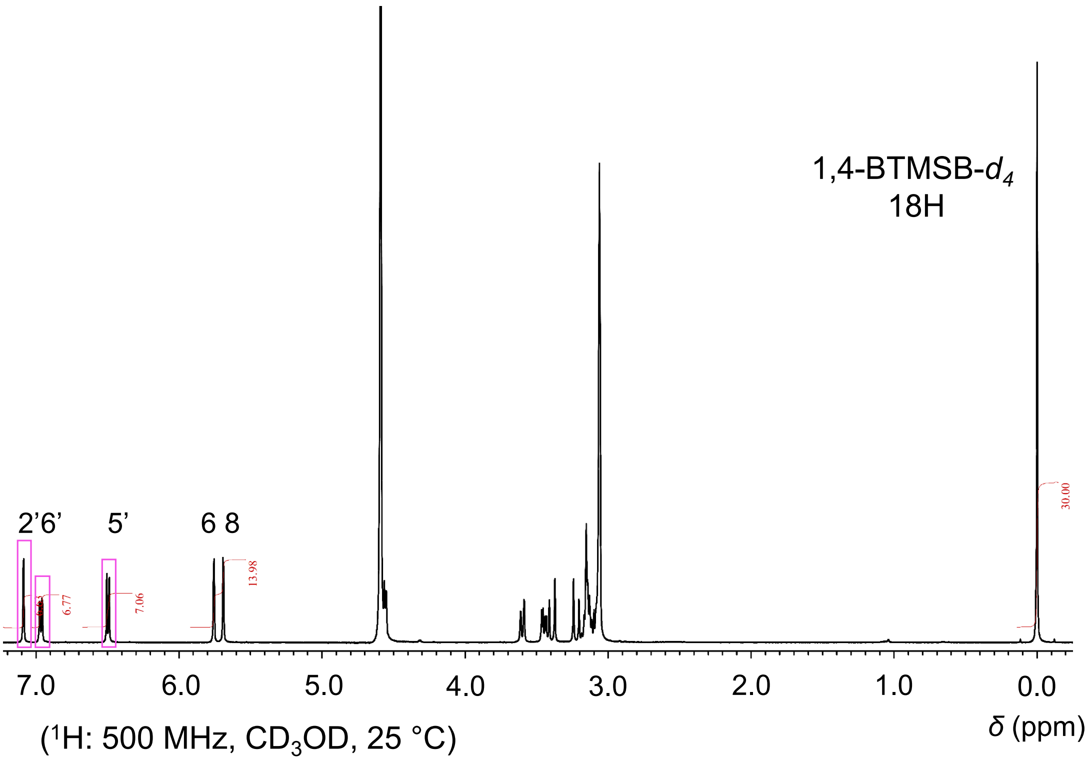

Figure S11. ^{1}H NMR spectrum of 2 with internal standard.

Table S5. Absolute purity of 2.

Entry	Purity (%)	Average (%)	RSD (%)
1	89.6		
2	88.2	88.7	0.71
3	88.3		

9. Quantitative NMR analysis (qNMR)-2: method using external standard

Purity assessments were performed by external calibration quantitative NMR (EC-qNMR) methodology employing the principle of reciprocity.[6-9] The samples were weighed and transferred to an empty vial. To the vial appropriate deuterated solvent was added and dissolved, then, the solution (0.6 mL) was transferred to the NMR tube (5 mm i.d.) and the tube flame-sealed. Cy3G (**1**) was dissolved in 5%TFA*d*-CD_3OD and DSS-d_6 was dissolved in CD_3OD. All experiments for EC-qNMR analysis were performed operating with JEOL JNM-ECZ600 at 599.67231 MHz equipped with a 5-mm Royal probe. The probe temperature was regulated at 25 ºC (298 K). The NMR operating software used was JEOL Delta v5.3.1. After the sample loading, shimming was done, then the probe was automatically tuned and matched. To determine 90PW, nutation experiments were performed by changing the values of PW (1, 5, 9, 13, 17, 21, 25, 29, 33 μsec), respectively. The number of scans (NS) for each PW was set to 1. The D_1 was set to 30 sec. Based on the spectra, 90PW was automatically calculated in curve analysis "Nutation Analysis" mode on Delta software. For EC-qNMR of samples, ^{13}C decoupled acquisition of the ^1H spectrum was performed by using "single_pulse_dec.jxp" as the original pulse sequence. MPF8 was selected as the ^{13}C decoupling modulation. The MPF8 was applied only during acquisition time (AQ). The NS was set to 16 and the dummy scans (ds) was set to 4. The relaxation delay (D_1) was set to 50 sec for maintaining the pulse repetition time (T_r) [AQ + D_1], is at least ten times the longest T_1 for the signals interested. The AQ was unified to 8.72 sec by setting the number of data points to 131,072 at a sufficient spectral width of 15,024 Hz. The 1,502 Hz at both ends were clipped; remaining 12,019 Hz was subjected to processing for transformation into spectrum. The FID was zero filled to 524,288 points (4 times) without any window functions. Then the phase of the spectrum was adjusted manually, and then baseline corrected (Akima fitting). The processed spectrum was saved separately from the original FID. The processed spectra of analyte and external calibrant (EC) were subjected to "Quantitative Analysis" on Delta software. Signals were automatically integrated. Based on the integral values, 90PW, and sample concentrations, quantitative value of analyte was automatically calculated from equation 2. where the subscripts denote the substances, analyte (A) and calibrant (C); *Molar conc.*, concentration; *S*, absolute integral value; *H*, number of protons; *90PW*, 90° pulse width; *T*, temperature in Kelvin.

The EC-qNMR was applied to commercially available **1** and the isolated **1** by Yoshida laboratory of Nagoya University. The absolute purities obtained by EC-qNMR were summarized in Table S5. When each absolute purity was applied to the calibration curve in

HPLC analysis, the slopes of the commercially available **1** and the isolated **1** approximated (Figure S12). The results imply that an isolated compound having the purity lower than the reagent can be used as a calibrant for HPLC quantitation, as long as the calibration curve can be corrected by the absolute purity obtained by qNMR. Furthermore, it is also desirable to perform qNMR on commercially available reagents. The label of such reagent often shows the purity as the area percentage; however, it is not the absolute value but a relative one. Indeed, qNMR revealed that commercially available reagent Cy3G (**1**) was less than 90% (84.5%, Table S6). Therefore, qNMR can represent one of the promising tools for getting reliable chromatographic outcomes.

Equation S2

$$Molar\ Conc._{\mathrm{A}} = Molar\ Conc._{\mathrm{C}} \times S_{\mathrm{A}}/S_{\mathrm{C}} \times H_{\mathrm{C}}/H_{\mathrm{A}} \times 90PW_{\mathrm{A}}/90PW_{\mathrm{C}} \times T_{\mathrm{C}}/T_{\mathrm{A}}$$

Table S6. Absolute purity of 1 obtained by EC-qHNMR.

Sample	Position (ppm)	Group	Absolute purity	Average
Reagent **1**	8.984	H-4	83.8%	84.5%
	8.218	H-6'	85.1%	
	8.007	H-2'	84.2%	
	6.982	H-5'	84.8%	
Isolated **1**	8.984	H-4	72.9%	72.9%
	8.217	H-6'	73.1%	
	8.008	H-2'	72.4%	
	6.982	H-5'	73.2%	

Signals derived from H-6 and H-8 were not sitable for quantitative analysis due to the hydrogen-deuterium (H→D) exchamge.[10] Absolute purities were calculated as Cl-salt.

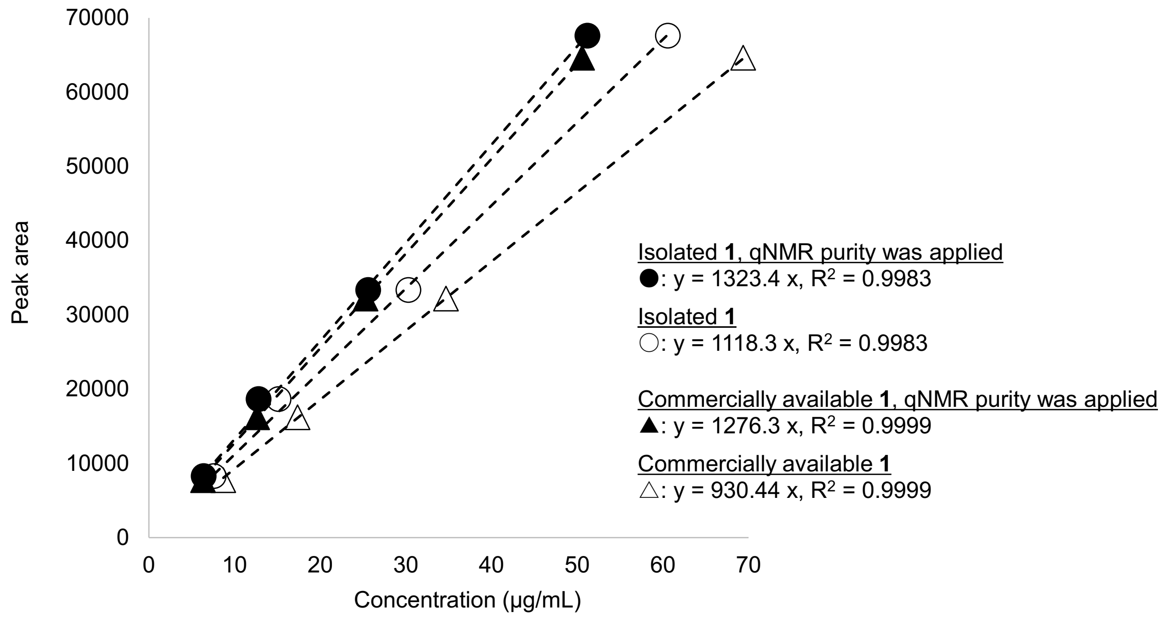

Figure S12. Calibration curves of the commercially available Cy3G (1) and the isolated 1 in HPLC/PDA analysis.

The following HPLC/PDA conditions were used: instrument, ACQUITY UPLC H-Class system (Waters); column, ACQUITY UPLC BEH C18 (2.1 × 150 mm, 1.7μm, Waters); column temperature, 40 ºC; mobile phase, 1.5% phosphoric acid: acetonitrile = 98 : 2 (0 min) → 50 : 50 (15 min); flow rate, 0.4 mL/min; detection, 516 nm.

10. Quantitative analysis of Cy3G (1) by HPLC.

Three immature beans at different stages and different exposure treatments were weighed and frozen with liq. N_2. The extraction solvent (3% TFA-50% CH_3CN aq, 3.0 mL/g FW) was added to the beans, and the sample was allowed to stand at room temperature in the dark for 24 h. The extract was then diluted five-fold with 3% TFA-H_2O, subjected to cartridge filtration (pore size: 0.45 μm), and filtrate was analyzed by HPLC (Condition: Table S1). The chloride salt of **1** was weighed precisely in triplicate, and the individual samples were diluted with 3% TFA-MeOH. Each sample was analyzed by HPLC (Condition: Table S1) in triplicate (Table S6), and the obtained peak areas detected with 530 nm were used for the construction of the calibration curve (Fig. S13).

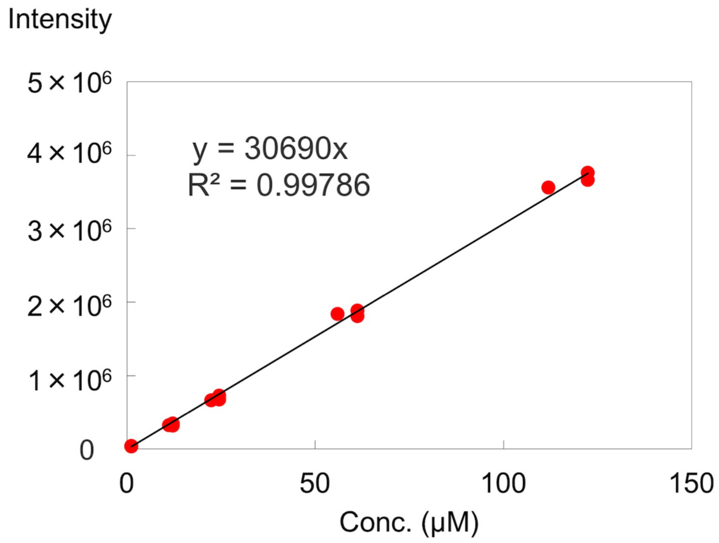

Figure S13. Calibration curve of 1.

11. Quantitative analysis of 2-F3G (2) by HPLC.

Three immature beans of different stages and different exposure treatments were weighed, frozen with liq. N_2, and extracted with 50% aq. CH_3CN (3.0 mL/g FW). The extract was then diluted five-fold with H_2O, filtered through a cartridge (pore size: 0.45 µm), and analysis of the filtrate carried out by HPLC (Condition Table S7). For calibration, the dried **2** was weighed precisely in triplicate, and the individual samples were diluted with MeOH. Each sample was analyzed by HPLC in triplicate (Table S7), and the obtained peak areas detected with 280 nm were used for the construction of the calibration curve (Fig. S14).

Table S7. HPLC condition of analysis of 2.

Column	RPAQUEOUS-AR-3, 2.0 mm i.d.×150 mm
Mobile phase	A: 0.5%TFA-10%CH_3CN aq.
	B: 0.5%TFA-90%CH_3CN aq.
Gradient condition	Linier gradient elution
0 min	A: 100%
1 min	A: 94%
10 min	A: 94%
13 min	A: 50%
14 min	A: 0%
17 min	A: 0%
Flow rate	0.2 mL/min
Detection	Photodiode array 200-650 nm
Temperature	40 °C

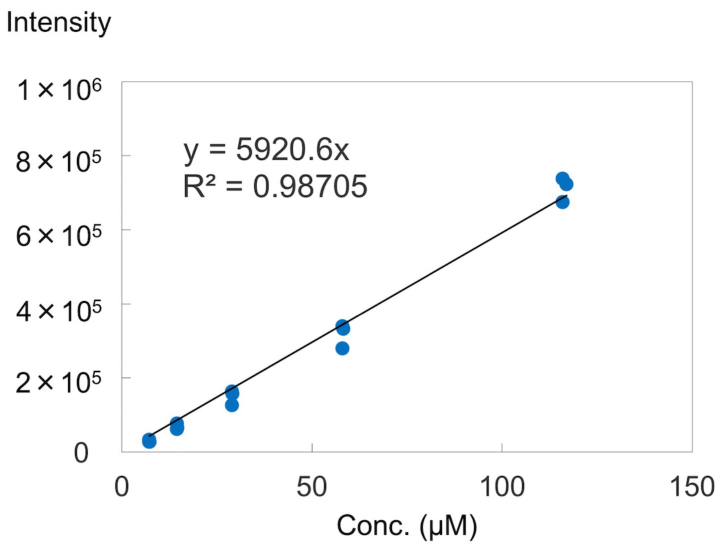

Figure S14. Calibration curve of 2.

12. Preparation of crude protein extract from immature seed coat

Ccrude protein extraction was carried out according to the previous report.[11] Immature seed coat of *G. max.* cv. Iwaikuro (5.0 g) was frozen with liq. N_2 and crushed using motor and pestle, then put into a tube. To the tube 5.0 mL of the extraction solution composed of ascorbic acid (10 mM), dithiothreitol (5 mM), phenylmethanesulfonyl fluoride (1 mM), ethylenediaminetetraacetic acid (1 mM), 3-[(3-Cholamidopropyl)dimethylammonio]-propanesulfonate (0.1% w/v), 2-mercaptoethanol (0.1% w/v), polyvinylpyrrolidone (5% w/v), DOWEX lx2 50-100 (10% w/v) in 100 mM Tris-HCl (pH 7.5) was added and kept for 1 min at 4 °C, then, centrifuged (20,000*g* 30 min, 4 °C) to be obtained a supernatant as crude protein extract. For negative control the crude protein extract was heated at 100 °C for 1 h, then, centrifuged (20,000*g* 30 min, 4 °C) to be obtained a supernatant as heated crude protein extract. The protein content in the both crude extract and heated crude extract was analyzed by using Pierce BCA Prtein Kit (Thermo Fisher Scientfic, Waltham, USA).

13. *In vitro* conversion of 5,7,3',4'-tetrahydroxyflav-2-en-3-ol 3-*O*-glucoside (2F3G, 2) to cyanidin 3-*O*-glucoside (Cy3G, 1) by addition of crude extract

In vitro conversion of **2** to **1** according to the procedure for ANS reported by Saito et al[12] was carried out. To an assay medium composed of crude protein extract (10 μg/μL), dithiothreitol (5 mM), NaCl (200 mM), maltose (10 mM), 2-oxoglutaric acid (1 mM), sodium ascorbate (4 mM), and $FeSO_4 \cdot 7H_2O$ (0.4 mM) in phosphate buffer (20 mM, pH 7.0), **2** (1 mM) was added and kept at 30 °C for 48 h. The reaction mixture was diluted with 3% TFA aq. for 5 times, then, analyzed by HPLC. The same assay at pH 5.0 (20 mM acetate buffer) was carried out.

Table S8. HPLC condition for analysis of 1 and 2.

Column	RPAQUEOUS-AR-3, 2.0 mm i.d.×150 mm
Mobile phase	A: 0.5%TFA-10%CH_3CN aq.
	B: 0.5%TFA-90%CH_3CN aq.
Gradient condition	Linier gradient elution
0 min	A: 90%
1 min	A: 94%
5 min	A: 94%
14 min	A: 60%
Flow rate	0.2 mL/min
Detection	Photodiode array 200-650 nm
Temperature	40 °C

At pH 7.0, **2** was not converted most and remained after 48 h incubation w/wo crude protein. However, at pH 5.0 **1** increased corresponding decrease of **2** (Fig. S15).

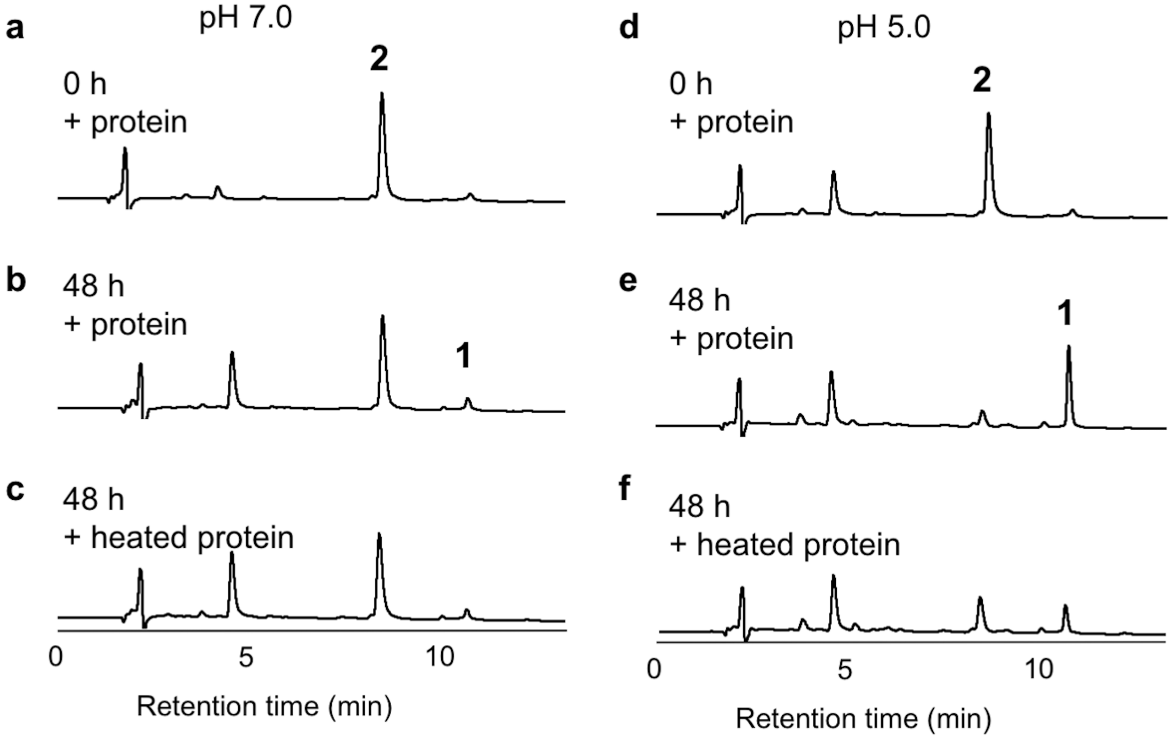

Figure S15 Conversion of 2 to 1 by addition of crude protein extract analyzed by HPLC at pH 7.0 (a–c) and at pH 5.0 (d–f).

HPLC chromatogram of reaction mixture at pH 7.0 at 0 h (**a**), with crude protein 48 h (**b**) and heated crude protein 48 h (**c**). HPLC chromatogram of reaction mixture at pH 5.0 at 0 h (**d**), with crude protein 48 h (**e**) and heated crude protein 48 h (**f**).

14. *In vitro* conversion of 5,7,3’,4’-tetrahydroxyflav-2-en-3-ol 3-*O*-glucoside (2F3G, 2) to cyanidin 3-*O*-glucoside (Cy3G, 1) by Ferrous ion without crude protein

In vitro conversion of **2** (1 mM) to **1** without crude protein in an assay medium composed of sodium ascorbate (4 mM), and $FeSO_4 \cdot 7H_2O$ (0.4 mM) in acetate buffer (20 mM, pH 5.0) and phosphate buffer (20 mM, pH 7.0) at 30 °C was carried out. The reaction mixture was diluted with 3% TFA aq. for 5 times, then, analyzed by HPLC with the elution condition as shown in Table S8.

15. Statistical Analysis

The quantitative analyses of Cy3G and 2-F3G in the immature black soybean sample were performed in triplicate. The data were analyzed by one-way ANOVA with the post hoc Scheffe test. The significant differences are indicated in the figures using different characters as explained in the figure legends.

16. References

1. Teppabut, Y., Oyama, K. I., Kondo, T. & Yoshida, K. Change of Petals' Color and Chemical Components in *Oenothera* Flowers during Senescence. *Molecules* 23, 1698 (2018).
2. Yoshida, K. et al. Structural Analysis and Measurement of Anthocyanins from Colored Seed Coats of *Vigna*, *Phaseolus*, and *Glycine* Legumes. *Biosci. Biotechnol. Biochem.* 60, 589–593 (1996).
3. Yoshida, K., Kond, T. & Oyama, K-I. The method of producing anthocyanidins and related flavenols. JPN Patent. No. 5382676, 2014.
4. Oyama, K-I. et al. Conversion of flavonol glycoside to anthocyanin: an interpretation of the oxidation-reduction relationship of biosynthetic flavonoid-intermediates. *RSC Adv.* 9, 31345-31439 (2019).
5. Uchiyama, N., Masada, S., Hosoe, J., Hakamatsuka, T. & Goda, Y. Determination of absolute purities of commercial agents used for the quantification of functional substances by quantitative NMR analysis. *Jpn. J. Food. Chem. Safety* 24, 125-130 (2017).
6. Hoult, D.I.; Richards, R.E. The signal-to-noise ratio of the nuclear magnetic resonance experiment. *J. Magn. Reson.* 1976, *24*, 71−85.
7. Hoult, D.I. The principle of reciprocity in signal strength calculations — a mathematical guide. *Concepts Magn. Reson.* 2000, *12*, 173−187.
8. Burton, I.W.; Quilliam, M.A.; Walter, J.A. Quantitative ^{1}H NMR with external standards: use in preparation of calibration solutions for algal toxins and other natural products. *Anal. Chem*. 2005, *77*, 3123−3131.
9. Wider, G.; Dreier, L. Measuring protein concentrations by NMR spectroscopy. *J. Am. Chem. Soc.* 2006, *128*, 2571−2576.
10. Jordheim, M.; Fossen, T.; Songstad, J.; Andersen, Ø.M. Reactivity of anthocyanins and pyranoanthocyanins. Studies on aromatic hydrogen-deuterium exchange reactions in methanol. *J. Agric. Food Chem.* 2007, *55*, 8261−8268.

11. Ohgami, S., et al., Volatile glycosylation in tea plants: Sequential glycosylations for the biosynthesis of aroma b-primeverosides are catalyzed by two *Camellia sinensis* glycosyltransferases. *Plant Physiol*. 168, 464-477 (2015).

12. Saito, K., Kobayashi, M., Gong, Z., Tanaka, Y. & Yamazaki, M. Direct Evidence for Anthocyanidin Synthase as a 2-Oxoglutarate-Dependent Oxygenase: Molecular Cloning and Functional Expression of CDNA from a Red Forma of *Perilla Frutescens*. *Plant J.* 17, 181–189 (1999).
